# Deciphering Non-coding RNAs in Cardiovascular Health and Disease

**DOI:** 10.3389/fcvm.2018.00073

**Published:** 2018-07-02

**Authors:** Anindita Das, Arun Samidurai, Fadi N. Salloum

**Affiliations:** Pauley Heart Center, Division of Cardiology, Department of Internal Medicine, Virginia Commonwealth University, Richmond, VA, United States

**Keywords:** long non-coding RNA, microRNA, myocardial infarction, hypertrophy, atherosclerosis, diabetic cardiomyopathy

## Abstract

After being long considered as “junk” in the human genome, non-coding RNAs (ncRNAs) currently represent one of the newest frontiers in cardiovascular disease (CVD) since they have emerged in recent years as potential therapeutic targets. Different types of ncRNAs exist, including small ncRNAs that have fewer than 200 nucleotides, which are mostly known as microRNAs (miRNAs), and long ncRNAs that have more than 200 nucleotides. Recent discoveries on the role of ncRNAs in epigenetic and transcriptional regulation, atherosclerosis, myocardial ischemia/reperfusion (I/R) injury and infarction (MI), adverse cardiac remodeling and hypertrophy, insulin resistance, and diabetic cardiomyopathy prompted vast interest in exploring candidate ncRNAs for utilization as potential therapeutic targets and/or diagnostic/prognostic biomarkers in CVDs. This review will discuss our current knowledge concerning the roles of different types of ncRNAs in cardiovascular health and disease and provide some insight on the cardioprotective signaling pathways elicited by the non-coding genome. We will highlight important basic and clinical breakthroughs that support employing ncRNAs for treatment or early diagnosis of a variety of CVDs, and also depict the most relevant limitations that challenge this novel therapeutic approach.

## Introduction

The discovery of the new class of ribonucleic acids (RNAs), namely non-coding RNAs (ncRNAs), revolutionized our knowledge about the epigenetic, post-transcriptional, and post-translational modification of gene expression in the regulation of tissue homeostasis in health and disease. Recent advances in the field of genomics enabled with technologies like next generation sequencing (NGS), ChIP RNA Seq, and transcriptome analysis have offered new perspectives and completely changed our understanding on small ncRNA molecules, once considered as “junk DNA.” Nearly 99% of the genome consists of non-coding DNA, whereas only 1% codes for functional proteins, which reflects the complexity and importance of ncRNAs in controlling gene expression. Regulatory ncRNAs such as microRNAs (miRNAs) and long non-coding RNAs (lncRNAs) have drastically impacted research in multiple fields, including cardiovascular diseases (1–3) (CVD), diabetes (4, 5), and cancer (6, 7). The epigenetic regulation of these ncRNAs, like miRs, plays a very significant role both in the early stage of development and during the pathogenesis of heart disease (3, 8, 9).

Cardiovascular disease (CVD) remains the leading cause of mortality and morbidity worldwide (10, 11). CVD is a broad term used to describe abnormalities affecting the heart and its associated circulatory system, such as atherosclerosis, myocardial ischemia/reperfusion (I/R) injury and infarction (MI), hypertension, and arrhythmias. The occurrence of risk factors, such as diabetes, obesity and advanced age leads to substantial complications of CVD ultimately leading to heart failure (HF). Although current management has improved survival in patients with CVD, such therapies do not fully address the underlying cause and, as a result, HF progresses. This highlights an urgent need to develop novel diagnostic and therapeutic approaches to alleviate symptoms, improve cardiac function and quality of life, slow disease progression, and reduce mortality in patients with CVD and HF (12, 13). Emerging concepts based on genomic information have redesigned diagnostic and treatment strategies, enabling early detection of abnormalities and offering hope for more effective treatment options. This review aims to provide the readers with an updated summary on the role of ncRNAs in cardiovascular physiology and pathophysiology, with emphasis on MI, atherosclerosis, diabetic cardiomyopathy, and HF. We will also review the potential utility of ncRNAs as a therapeutic option and describe some of the current limitations.

## Non-coding RNAs: classification and mechanisms

Non-coding RNAs are classified into several types based on their length and mechanism of gene regulation. This includes miRNAs (<25 nt), lncRNA (>200 nt), piRNAs (RNA-protein complexes), and siRNAs (double-stranded RNA, ~20–25 nt), which are among the most thoroughly investigated ncRNAs. Even though regulation of gene expression is the primary function of these ncRNAs, they each achieve this goal via different methods.

### miRNA biosynthesis and function

**Figure 1:**
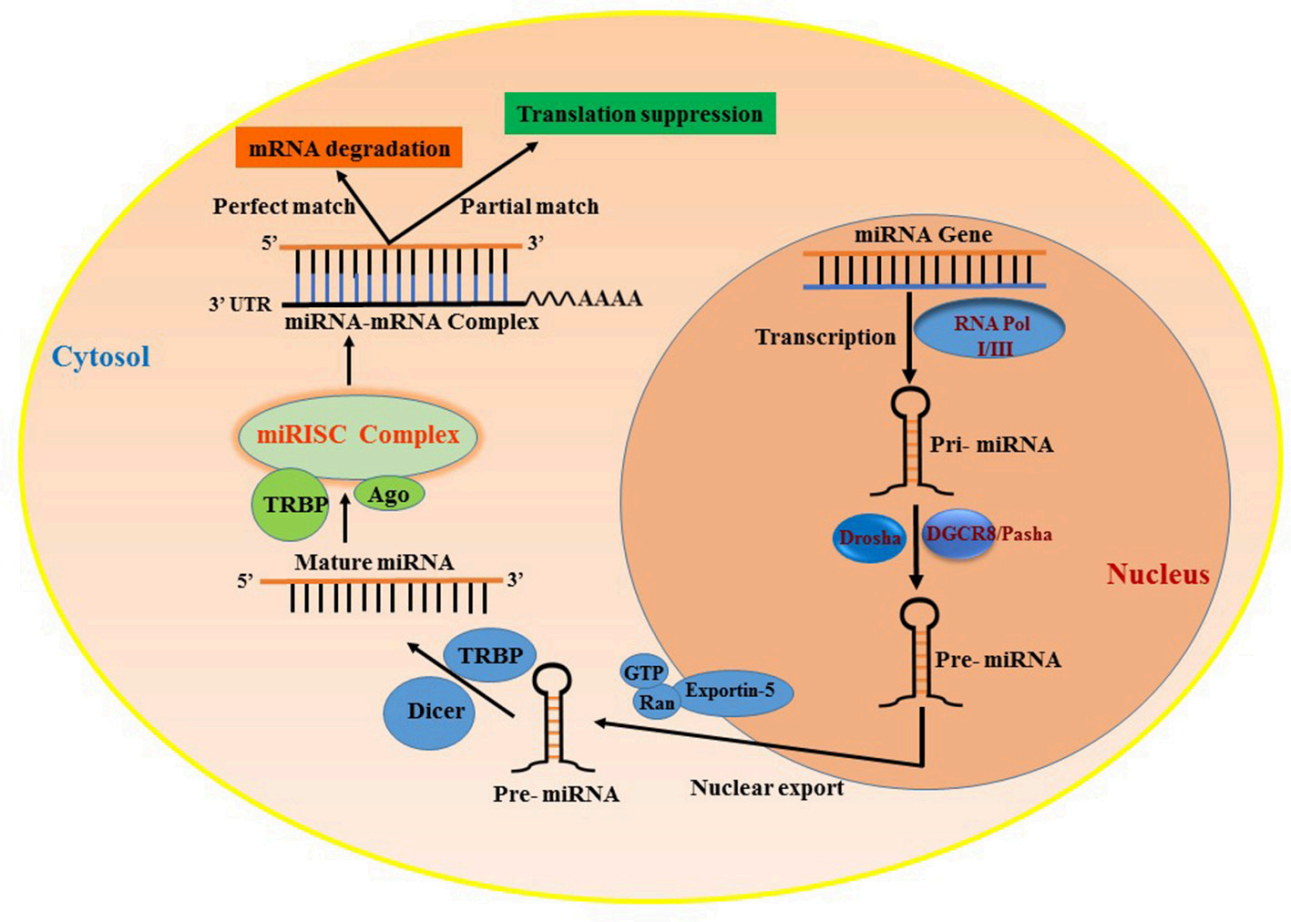
Biogenesis of miRNAs and their mode of transcript suppression. RNA-Pol, RNA polymerase; Drosha; RISC, RNA induced silencing complex; DGCR8, diGeorge syndrome critical region-8; GTP, guanosine triphosphate; Ras, RAs-related nuclear protein; TRBP, human immunodeficiency virus trans-activating response RNA-binding protein; Ago2, Argonaute 2.

MicroRNAs (miRNAs) are small ncRNAs that are approximately 18–25 nt in length and regulate gene expression by binding to the 3′UTR of mRNA, leading to either degradation or translational suppression of its target mRNA (14, 15). The first miRNA was discovered in 1993 in a study examining developmental regulatory genes in *Caenorhabditis elegans* (14). miRNAs are among the most abundantly occurring ncRNAs that are widely distributed in several tissues and are conserved among species. There are more than 2,500 miRNAs reported in the human genome (miRBase) (16) that are known to specifically regulate gene expression. Almost all miRNAs are transcribed from either introns or intergenic region, by the enzyme RNA polymerase II or in some cases RNA polymerase III into a hairpin precursor molecule called primary-miRNA. These primary-miRNAs (100 nt) undergo maturation process by the enzyme Drosha, which cleaves them to Pre-miRNAs (~70 nt) (17). Pre-miRNAs are later exported to the cytoplasm by Exportin-5. Once out of the nucleus, the pre-miRNA further undergoes cleavage to a mature double-stranded miRNA of 22 nt by the enzyme RNase III (18). The active strand of the mature miRNA binds to RNA-induced silencing complex (RISC) and interferes in the transactivation-responsive RNA-binding protein (TRBP) and Argonaute 2 (Ago2) and inhibits the 3′UTR of the target mRNA through base-pair interactions (19) and negatively regulates its expression. These steps are collectively illustrated in Figure 1. Each miRNA can have multiple targets and is primarily based on the presence of complementary binding sequence in the mRNA. The extent of sequence complementarity between the miRNA and mRNA determines whether the target mRNA is destined for complete degradation or translational inhibition. Nucleotide sequence (2–7) at the 5′ end of miRNA, which forms the seed region, is critical for the formation miRNA-mRNA binding complex. Also, miRNAs can be generated from both guide as well as passenger strands of the DNA and are denoted by 3-p or 5-p suffix. The opposite strand, often called the passenger strand due to its relatively lower levels in the steady state, is denoted with an asterisk (^*^) and is normally degraded. In some cases, miRNA generated from both strands are viable and are incorporated in RISC complex and become functional miRNAs that target different mRNAs (20, 21). The loci of miRNA are located at various genomic contexts and while the majority of them are found in the intronic region, they can also be encoded at coding transcripts and even in the exonic regions (22). Often, several miRNAs that belong to the same cluster are co-transcribed simultaneously, but may have their own individual function after undergoing a separate post-transcriptional regulation (23, 24). Apart from the aforementioned classical canonical miRNA biogenesis pathway, miRNA synthesis may also follow a non-canonical pathway to maturation (25, 26), where short introns are processed through splicing independent of Drosha/DGCR8 processing known as mirtron pathway (27–29). Several miRNAs such as endogenous short hairpin RNAs (30), small nucleolar RNAs (snoRNAs) (31), and tRNAs (31, 32) belong to this class of miRNAs. Some mirtrons are processed through simtron route, where Drosha is required but does not necessitate Drosha's binding partner DGCR8 or endonuclease (33). Both simtrons and mirtrons are capable of silencing target transcripts and are associated with the RISC complex as evidenced by their interaction with Argonaute proteins. Regardless of the differences in various miRNA biogenesis pathways, they all result in functional mature miRNAs. The existence of several mechanisms in the biogenesis of miRNAs further reflects the complexity of RNA processing.

### Long non-coding RNA

**Figure 2:**
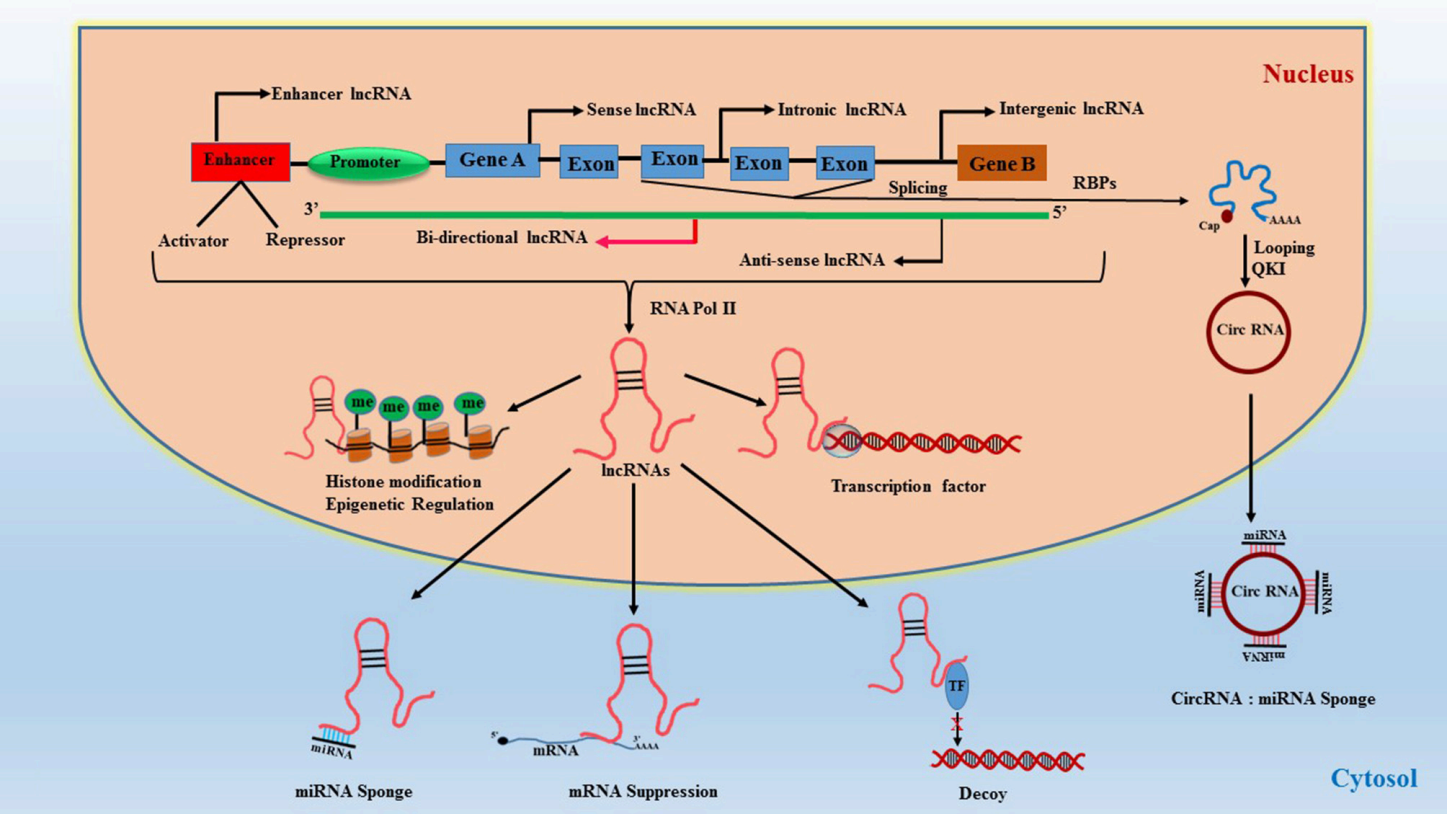
Classification of lncRNAs and their function. RBPs, RNA-binding proteins; QKI, quaking protein; Circ-RNA, circular RNA; TF, transcription factor.

Long non-coding RNAs (LncRNAs) represent a highly diverse group of regulatory ncRNAs with respect to their characteristics, localization, and mode of action. The lncRNAs are longer than 200 nucleotides in size and are regulators of gene expression both at the transcriptional as well as post-transcriptional levels (34). They are synthesized by RNA polymerase II as co-factors along with gene activation and contain poly-A tail end and 5′capping. They function as *cis* and *trans* acting elements for protein-coding DNA sequences and therefore are powerful epigenetic mediators (35, 36). Due to this functionality, they can act both as negative or positive effectors of gene expression. LncRNAs are synthesized similar to regular mRNA transcripts but lack a defined open reading frame (ORF). They also contain their own promoter elements and can be transcribed as part of the gene. Interestingly, they can also be regulated by miRNAs (37) which adds another layer of transcriptional regulation. LncRNAs contain complementary sequences to miRNAs and can act as miRNA sponge/decoy. Briefly, lncRNAs are classified based on their location in the genome, their length, proximity to protein-coding genes, association with DNA elements, mechanisms of action, and sub-cellular localization (nucleus or cytoplasm). They are broadly classified based on their genomic loci and function (34) (1) **Sense lncRNAs** are synthesized from exons of protein-coding genes utilizing the same promoter region of the gene (38, 39), (2) **Antisense lncRNAs** are aberrant transcripts synthesized from the opposite strand of protein-coding region (40, 41), (3) **Intronic lncRNAs** are generated from an intron of a protein-coding gene (39, 42), (4) **Intergenic lncRNAs**, also referred to as large intergenic (or intervening) non-coding RNAs (lincRNAs), are encoded between protein-coding genes and are transcribed independently (43, 44), (5) **Enhancer lncRNAs**, also known as enhancer RNA (eRNA), are synthesized from the transcription binding regions i.e., activator/repressor elements of a protein-coding gene (45), (6) **Circular** forms are RNAs where the 3′-5′ ends are covalently enclosed to create a circular loop derived from splicing of a protein-coding gene (46, 47), and (7) **Bidirectional** transcripts are transcribed from the same promoter as coding genes, but in the opposite direction (48). The classification and function of lncRNAs are illustrated in Figure 2.

A plethora of lncRNAs have been identified, but their function and regulation are not yet completely understood (49, 50). Nevertheless, several studies report a role for lncRNA in organ development and differentiation, and also in human diseases (51–53) especially in CVD (13, 54). Apart from direct gene silencing, lncRNAs regulate histones and influence epigenetics through modulation of DNA methylation at CpG dinucleotides, which is critical for the repression of genes (55).

### siRNA

Discovered in 1999 (56), small interfering RNA or short interfering RNA (siRNA) is one of the most extensively exploited ncRNA in RNA interference therapies. siRNAs are closely related to miRNAs in terms of size and biogenesis, but slightly differ in their mechanism of RNA silencing. Unlike miRNAs, which are single-stranded RNA, siRNAs are double-stranded RNA (dsRNA) and are approximately 20–24-bp in size with a well-defined structure (57). siRNAs bind their target with 100% complementarity in the sequence and typically cleave mRNA before entering the translation process. Therefore, they are very highly specific in annealing to their target. They are also processed in a much similar fashion to miRNAs, synthesized by RNA pol III, cleaved by the enzyme Dicer and induce mRNA degradation via RISC formation (58–60). Due to their stability and the convenience of generating synthetic dsRNA that can be easily introduced exogenously into cells, siRNAs are widely used in gene therapy to silence mRNA transcripts.

### Piwi-interacting RNA (piRNA)

First identified in 1997 (61–64), piRNAs are ncRNAs that are altogether different from miRNAs and lncRNAs. piRNAs are mostly found in the genome as clusters and range in size from 25 to 30 nucleotides (65, 66). piRNAs interact with piwi (P-element induced wimpy testis) proteins of Argonaute family, thereby leading to the formation of silencing ribonucleoprotein complex, which recognizes and silences the complementary sequence (67, 68). The piRNA/PIWI complex primarily functions as epigenetic silencer by targeting transposable elements (TEs) in both germline and gonadal somatic cells and it regulates the process of transcription itself rather than transcripts (69).

**Figure 3:**
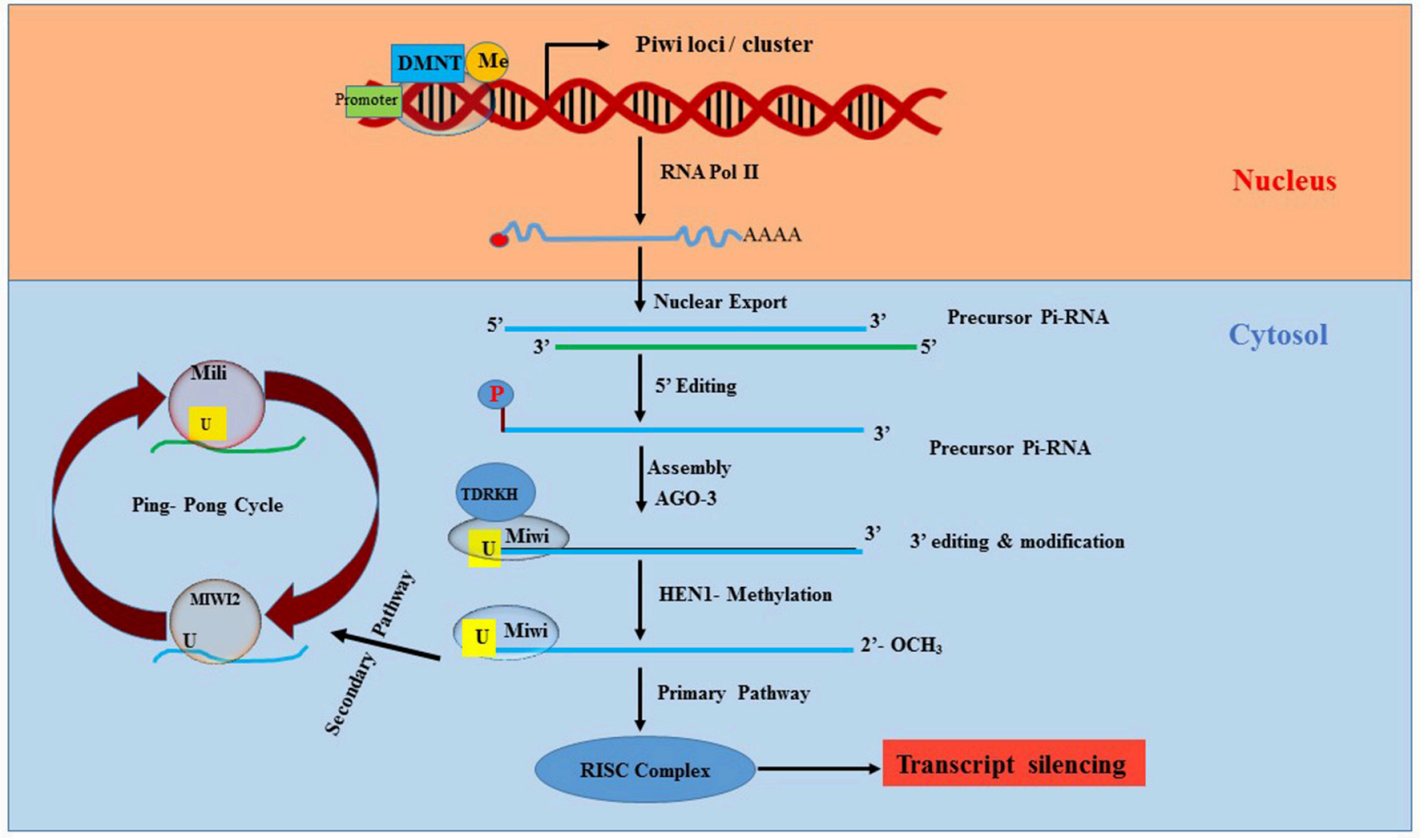
Synthesis and processing of Pi-RNA. Piwi, P-element Induced WImpy testis in Drosophila; piRNA, Piwi protein interaction RNA; DNMT, DNA methyltransferase; AGO3, Argonaute 3; HEN1, HUA ENHANCER 1; RISC, RNA induced silencing complex; Miwi, Mouse homolog of PIWI; TDRKH, Tudor domain to arginine methylated Miwi.

The mechanism of piRNA biogenesis and function is not yet completely clear and rather very complex. However, reports suggest that piRNAs regulate mobile sequences in the genome by canonically involving endonucleolytic cleavage of the target sequence after complementary base-pair recognition through piRISC (piRNA-induced silencing complex) (70), as illustrated in Figure 3. There are more than 50,000 piRNA sequences identified in the murine genome, but inconsistencies in the sequence homology between species makes it difficult to determine their function. Research to elucidate the function of piRNAs is still in its early stages. Nevertheless, piRNAs are widely accepted to play a strong role in epigenetics via regulation of TEs. Since TEs are important for genetic diversity and genome instability, any abnormalities in TEs can lead to gene deregulation, chromosome rearrangement and gene mutations causing cancer and genetic diseases (71, 72).

## MicroRNAs and cardiovascular diseases

The most extensively studied ncRNAs are miRNAs, which are abundantly present in many cardiac cell types including fibroblasts, endothelial cells (ECs), and cardiomyocytes. They play a significant role in several cellular processes like proliferation, apoptosis, autophagy, and cell metabolism. Dysregulation of individual or cluster of miRNAs is linked to the pathogenesis of heart diseases and its risk factors such as diabetes, hypertension, atherosclerosis, myocardial I/R injury, and HF (8, 73). The role of miRNAs in several CVD has been well-established by taking advantage of genetically modified animal models and *in vitro* cell lines, and utilizing miRNA mimics and inhibitors (antagomiRs). Moreover, the aberrant expression of miRNAs and subsequent impact on cellular signaling pathways are well-documented in the literature (74, 75).

### Adverse cardiac remodeling and heart failure

**Figure 4:**
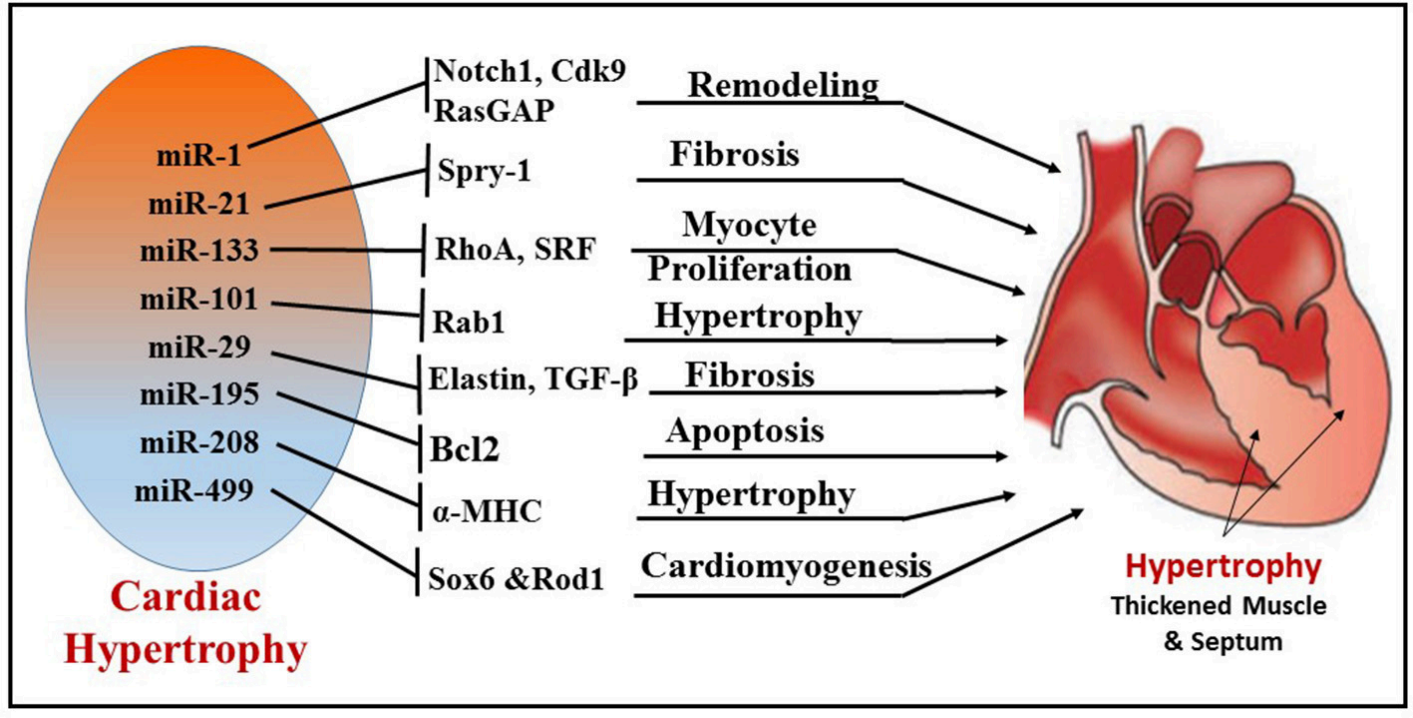
Illustration depicting the different miRNAs and their targets, which regulate corresponding cellular functions, like fibrosis, myocyte proliferation, apoptosis, cardiomyogenesis, and remodeling during cardiac hypertrophy.

Cardiac remodeling is a progressive reactive phenomenon to myocardial injury that involves cellular, molecular and interstitial changes that manifest physiologically and ultimately lead to HF. Significant dysregulation of miRNA expression has been implicated during cardiac hypertrophy and HF (8, 73) (Figure 4). Preliminary evidence for the role of miRNA in myocardial development was reported from studies using Drosophila, where miRNA-1 (miR-1) was identified to regulate differentiation of cardiac and somatic muscle progenitors through Notch 1 receptor (76). After aortic constriction-induced hypertrophy in a mouse model, the muscle-specific miR-1 was significantly downregulated, plausibly through a serum response factor (SRF)-dependent mechanism (73). Overexpression of miR-1 inhibited its *in silico*-predicted, growth-related targets, including Ras GTPase-activating protein (RasGAP), cyclin-dependent kinase 9 (Cdk9), fibronectin, and Ras homolog enriched in brain (Rheb), in addition to protein synthesis and cell size. In this context, more supportive evidence was reported using cardiac muscle-specific targeted deletion of miR-1-2 in mouse, demonstrating a role of miR-1 in cardiac morphogenesis and cell-cycle control (77). Two mature miRNAs, miR-1 and miR-133, are derived from the same miRNA polycistron and transcribed together during development, but have distinct roles in modulating skeletal muscle proliferation and differentiation in cultured myoblasts (78). miR-1 promotes myogenesis by targeting histone deacetylase 4 (HDAC4), a transcriptional repressor of muscle gene expression, whereas miR-133 enhances myoblast proliferation by repressing SRF, a positive regulator of cardiac growth and HF. Among the miRs that were down-regulated during cardiac hypertrophy, both miR-1 and miR-133 have been prominently repressed in the left ventricle and atria in murine models as well as human subjects with cardiac hypertrophy (79). Overexpression of miR-133 or miR-1 inhibited cardiac hypertrophy. This notion was also confirmed in studies using antagomiR-133 showing sustained cardiac hypertrophy via RhoA, a GDP-GTP exchange protein target of miR-133. In contrast, another study demonstrated that overexpression of miR-1 in mouse cardiac progenitors has a negative effect on proliferation, where it targets the Hand transcription factor Hand2, which is involved in myocyte expansion (80).

A novel antifibrotic miRNA, miR-101, was found to be frequently downregulated in hypertrophic and post-infarcted hearts (81). MiR-101 plays a significant role in hypertrophy by regulating ras-related protein-1A (Rab1A), a member of the Rab family of small GTPases and an important regulator of cardiac hypertrophy (82). Expression of miR-101 in cardiomyocytes was downregulated in both the transverse abdominal aortic constriction rat model and angiotensin II (AngII)-induced hypertrophy. In addition, overexpression of miR-101 significantly suppressed AngII-induced cardiac hypertrophy by targeting Rab1A. In contrast, the inhibition of miR-101 expression promoted cardiac hypertrophy. The expression of miR-29 cluster (miR-29a, 29b, and 29c) inhibits the expression of targets involved in the extracellular matrix production and fibrosis (83). Moreover, the miR-29 family also controls pro-fibrotic genes such as elastin. Recent evidence also suggests that TGF-β signaling, an important regulator of fibrogenesis and collagen deposition, is regulated by several miRs including miR-29b, miR-26, miR-101a, and miR-24 miR101a (81, 84–86).

Overexpression of specific miRNAs evokes morphological changes in cardiomyocytes, which subsequently leads to ventricular hypertrophy and HF in humans (87). miRNA expression in idiopathic end-stage failing human hearts showed increased expression of miR-23a, miR-24, miR-125b, miR-195, miR-199a, and miR-214 (8). In addition, cardiac overexpression of miR-195 resulted in pathological cardiac remodeling and HF in transgenic mice. Other miRNAs have also been identified as important players in cardiac hypertrophy. For instance, cardiac-specific miR-208 encoded by an intron of the α-MHC (myosin heavy chain) gene is required for cardiomyocyte hypertrophy, fibrosis, and expression of β-MHC in response to stress and hypothyroidism (88). Moreover, the overexpression of miR-212 and miR-132 directly targets the anti-hypertrophic and pro-autophagic FoxO3 transcription factor and leads to cardiac hypertrophy and HF by inducing pro-hypertrophic calcineurin/NFAT signaling (89).

Several expression profile studies identified one of the most abundantly expressed miRNAs, namely miR-21, in murine and human hypertrophic and failing hearts. MiR-21 plays a crucial role in cardiac fibrosis and hypertrophy (90). Multiple studies reported that increased expression of miR-21 in fibroblasts of the failing heart induces the extent of interstitial fibrosis and cardiac hypertrophy by augmenting ERK–MAP kinase activity via inhibition of sprouty homolog 1 (Spry1) (91) or enhancing matrix metalloproteinase-2 (MMP-2) via PTEN (phosphatase and tensin homolog) pathway (92). Recently, miRNAs have emerged as regulators of intercellular communication in cardiac tissue (93). Bang et al. identified a high abundance of miR-21-3p (miR-21^*^) in cardiac fibroblast-derived exosomes as a paracrine signaling mediator that promotes cardiomyocyte hypertrophy by targeting sorbin and SH3 domain-containing protein 2 (SORBS2), PDZ and LIM domain 5 (PDLIM5). On the other hand, an antihypertrophic effect of miR-21 was also reported in transverse aortic constriction (TAC) and Ang II-treated mice (94). Overexpression of miR-21-3p suppressed TAC- and Ang II-induced cardiac hypertrophy by targeting histone deacetylase-8 (HDAC8) and modulating p-AKT/p-GSK3β pathway.

Apart from the direct involvement of certain miRNAs in hypertrophy and fibrosis, several other miRNAs were shown to be pro-hypertrophic. By targeting p53-induced nuclear protein (Tp53inp1), miR-155 regulates hypertrophy and cardiac remodeling (95). It was shown that miR-499 expression was upregulated in pressure overload-induced murine cardiac hypertrophy. This finding also correlated with increased expression of miR-499 in human failing and hypertrophied heart (94, 96, 97). Interestingly, miR-499 was responsible for the differentiation of cardiac stem cells (CSCs) into mature functional cardiomyocytes. To this end, overexpression of miR-499 in human cardiac stem cells (hCSCs) enhanced cardiomyogenesis by suppressing its target Sox6 and Rod1 (98).

### MicroRNAs in myocardial ischemia/reperfusion injury and infarction

**Figure 5:**
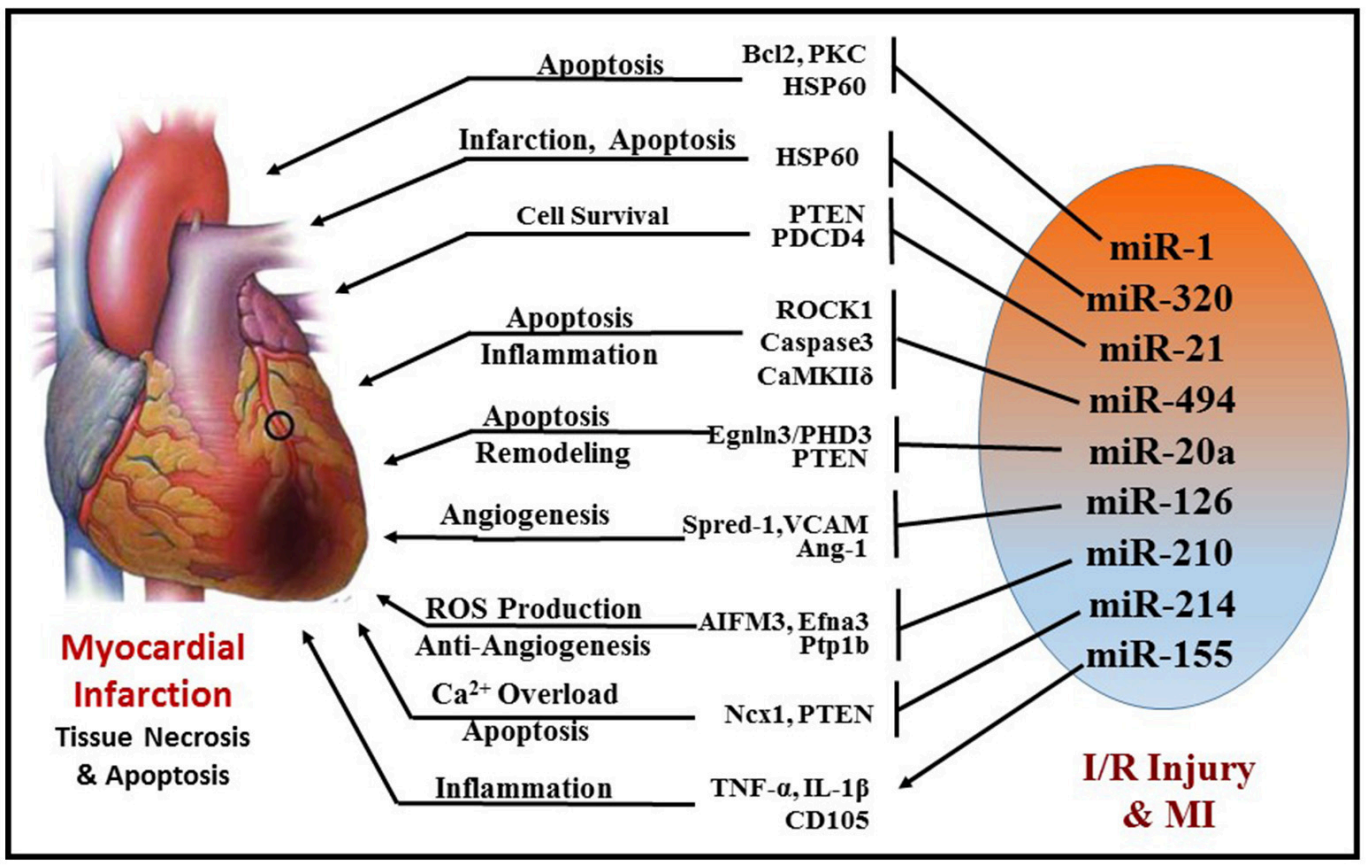
Schematic representation of miRNAs and their targets that are involved in cellular survival, apoptosis, angiogenesis, fibrosis, and inflammation during MI.

Ischemia/reperfusion (I/R) injury is a major cause of necrotic, apoptotic and autophagic cardiomyocyte death, all of which are highly regulated by miRNAs (Figure 5). Some of the prominent regulators of cardiomyocyte death are miR-1(99) miR-15b (100), miR-21 (101), miR-30b (102), miR-34a (103, 104), and miR-497 (105). Almost all these miRNAs target the anti-apoptotic gene BCL2 and negatively regulate them. MiR-1 was found to be markedly up-regulated during I/R injury and its expression level was inversely correlated to Bcl-2 expression in cardiomyocytes (99). In line with this finding, using miR-1 transgenic mice, Pan et al. revealed that miR-1 exacerbated cardiac I/R injury whereas knockdown of miR-1 with LNA-antimiR-1 alleviated cardiac I/R injury (99, 106). The same study also showed that inhibition of miR-1 can reduce apoptosis via regulating protein kinase C (PKC) and HSP60. A recent study also determined that myocardial I/R injury causes induction of miR-1 expression and subsequent downregulation of Bcl-2, which were reversed with hydrogen sulfide treatment resulting in attenuation of cardiomyocyte apoptosis (107). Functional studies indicate contrasting roles of miR-1 and miR-133 in the regulation of stress-induced cardiomyocyte survival, with a pro-apoptotic role of miR-1 and anti-apoptotic role of miR-133 (108). Post-MI, increased miR-1 represses multiple anti-apoptotic genes (i.e., Hsp60, Hsp70, IGF-1, and Bcl-2); whereas miR-133 negatively regulates a pro-apoptotic gene (i.e., Caspase-9) (99, 108, 109). Specifically, cardiac expression of miR-133 in patients who died following MI was significantly reduced in the infarcted areas of the heart compared to healthy adult hearts who died from non-cardiac causes (110). On the contrary, down-regulation of both miR-1 and miR-133 were reported in rat hearts after 30 min of coronary artery occlusion and 180 min reperfusion (111).

Another miRNA, miR-320, was shown to be differentially regulated in murine hearts after I/R injury both *in vivo* and *ex vivo*. Transgenic mice with cardiac-specific overexpression of miR-320 exhibited increased cardiomyocyte apoptosis and MI following I/R injury relative to the wild-type controls (112). Simultaneously, knockdown of endogenous miR-320 with antagomir-320 reduced infarct size and cardiomyocyte death. Using luciferase/GFP reporter assay, HSP20 (small heat-shock protein) was proven to be a target of miR-320. HSP20 plays a major role in cardioprotection against I/R injury by developing an adaptive response (113).

MiR-494 has also attracted considerable attention in the recent years. It is downregulated in failing human hearts and animal models of cardiac ischemia/hypertrophy. Transgenic mice with cardiac-specific overexpression of miR-494 displayed remarkable protection against myocardial I/R injury by reducing apoptosis and infarct size (114). Similarly, overexpression of miR-494 in cultured adult cardiomyocytes demonstrated inhibition of caspase-3 activity and reduced cell death upon simulated I/R. Furthermore, *in vivo* silencing of miR-494 aggravated I/R injury in mice. In this study, miR-494 was shown to target both pro-apoptotic (PTEN, ROCK1, and CaMKIIδ) as well as anti-apoptotic proteins (FGFR2 and LIF), but ultimately led to protection against myocardial I/R injury by activating AKT signaling.

miR-21 has also been shown to play a crucial role in attenuation of I/R injury by inducing several pro-survival signaling pathways in cardiomyocytes and targeting several pro-apoptotic genes, i.e., programmed cell death 4 (PDCD4), PTEN, and Fas ligand (FasL) (115–117). PTEN is essential for the activation of pro-survival AKT kinase pathway (118, 119) and inhibition of PTEN is known to limit infarct size. Transgenic mice with cardiac-specific over-expression of miR-21 exhibited suppression of ischemia-induced up-regulation of PTEN and FasL expression, increase in phospho-AKT, which collectively resulted in attenuation of infarct size and subsequent HF. Furthermore, ischemic pre-conditioning was shown to induce miR-21 in the mouse heart, which may mediate its cardioprotective effects against I/R injury (120, 121). Overexpression of miR-21 in rat hearts reduced myocardial infarct size with improved left ventricular remodeling 2 weeks after acute MI (122). Interestingly, induction of miR-21 by hydrogen sulfide was also proven to be beneficial in protecting the heart against MI and inflammasome activation (123). The protective effects of hydrogen sulfide were absent in cardiomyocytes treated with antagomiR-21 and in miR-21 knockout mice. Recently, miR-21 was also found to be pivotal in isoflurane-induced protection of cardiomyocytes against hypoxia/reoxygenation injury (124, 125). The beneficial effects of isoflurane against myocardial I/R injury were lost in miR-21 KO. This study demonstrates that Akt/NOS/mPTP pathway is involved in miR-21-mediated protective effect of isoflurane. Collectively, these studies indicate that miR-21 is induced in cardiomyocytes in the early phase of MI and contributes to myocardial protection. However, in the late phase of MI, induction of miR-21 predominantly in fibroblasts was shown to cause fibrosis and cardiac remodeling (125).

Another interesting player in myocardial I/R injury is miR-17-92 cluster consisting of family members, miR-17, miR-18a, miR-19a, miR-20a, miR-19b-1, and miR-92a, which is indispensable for cell proliferation and normal cardiac development (126, 127). Several studies have demonstrated that miR-17-92 is vital during cardiac morphogenesis and controls proliferation by targeting PTEN (128–130). Upregulation of miR-20a in neonatal rat cardiomyocytes following hypoxia/reoxygenation inhibits apoptosis, while its targeted knockdown induces cardiomyocyte apoptosis (131). The anti-apoptotic effect of miR-20a is mediated through targeted suppression of the pro-apoptotic factor Egnln3/PHD3 (prolyl hydroxylase 3). Moreover, cardiac-specific overexpression of miR-17–92 cluster also alleviates MI-induced injury and improves cardiac function in mice (128). A recent study revealed that miR-17-3p contributes to exercise-induced cardiac growth and protects against adverse ventricular remodeling after cardiac I/R injury (128, 132).

On the contrary, this cluster was also indicated to negatively affect angiogenesis (133) and the use of antagomiR-92a enhanced angiogenesis, improved left ventricular function, attenuated myocardial infarct size, and reduced apoptosis (134). This conflicting observation was in part due to the ablation of multiple miRNA members of the same cluster, as it is possible that members of the same cluster may have independent/conflicting functions by targeting different genes.

MiR-126 has been implicated as a protective miRNA, which is highly expressed in the heart. It functions as a promotor of new blood vessel formation by enhancing proangiogenic factor vascular endothelial growth factor (VEGF), inhibiting Sprouty-related protein-1 (Spred-1) and vascular cell adhesion molecule 1 (VCAM-1) and Angiopoietin-1 (Ang-1) (135–138). Consistent with this finding, Wang et al. demonstrated that targeted deletion of miR-126 in mice resulted in defective cardiac neovascularization with impairment of EC proliferation, migration, and angiogenesis following MI (135). Consistently, antogomiR-mediated silencing of EC specific miR-126 impaired angiogenesis following hindlimb ischemia (139). Moreover, miR-126 was also found in endothelial apoptotic bodies and was shown to mediate chemokine factor CXCL12 production leading to apoptosis during I/R injury. Similar to miR-126, miR-210 also promotes angiogenesis since its overexpression under normoxic conditions increased EC tubulogenesis and migration, whereas miR-210 inhibition in the presence of hypoxia decreased capillary-like formation, EC migration, survival, and induced apoptosis (140). Ephrin-A3 plays a crucial role in the development of the cardiovascular system and also in vascular remodeling (141). In response to hypoxia, miR-210 directly inhibits Ephrin-A3, which leads to stimulation of capillary-like formation and angiogenic response to ischemia (140). MiR-210 is also upregulated in hypoxic cardiomyocytes through Akt- and p53-dependent pathways and exerts cytoprotective effects by potentially reducing mitochondrial ROS production (142). Hu et al. demonstrated that induction of miR-210 rescues cardiac function after MI by upregulation of angiogenesis and inhibition of cellular apoptosis in the heart (143). Myocardial I/R injury is accompanied by mitochondrial calcium (Ca^2+^) overload, which contributes to mitochondrial dysfunction and cardiomyocyte death (144).

MiR-214 is yet another cardioprotective mediator against excessive Ca^2+^ overload in response to I/R injury. It targets sodium/calcium exchanger 1 (Ncx1)—a key regulator of Ca^2+^ influx, and influences several downstream effectors of Ca^2+^ signaling and cell death (145). MiR-214 protects the heart against I/R injury by inhibiting Ca^2+^ overload and cardiomyocyte death in response to I/R injury through its repression of NCX1, CaMKIIδ, CypD, and BIM. The beneficial role of miR-214 against I/R injury was further supported by reports demonstrating that genetic deletion of miR-214 in mice resulted in loss of calcium homeostasis, cardiac contractility, increased apoptosis and excessive fibrosis post-I/R (145). Alternatively, miR-214 can also inhibit PTEN and thus can regulate PI3-AKT mechanism during myocardial IR injury (146).

Recently, an interesting study showed miR-155 exacerbates I/R injury by enhancing the inflammation process in human muscle tissue (147). Data from this study showed that upregulation of miR-155 aggravates inflammatory response, leukocyte infiltration as well as cell death via induction of TNF-α, IL-1β, CD105, and Caspase3. Moreover, experiments conducted in miR-155 knockout mice displayed decreased inflammation upon I/R injury by regulation of suppressor of cytokine signaling 1 (SOCS-1) in a ROS-dependent manner (147).

Rane et al. reported that miR-199a is acutely down-regulated in cardiomyocytes during hypoxic conditions, which is obligatory for the rapid upregulation of its target, hypoxia-inducible factor HIF-1α, and hypoxia-induced apoptosis (148). Downregulation of miR-199 also induces hypoxia-induced pro-apoptotic genes like caspase −3,−6,−9, and−12 and FasL, AIF, and Bnip1. Replenishing miR-199a during hypoxia inhibits HIF-1α expression and reduces apoptosis. The same study also identified Sirt1 as another direct target of miR-199, which is responsible for downregulating prolyl hydroxylase 2 (PHD2)—required for stabilization of HIF-1α.

### MicroRNAs and atherosclerosis

**Figure 6:**
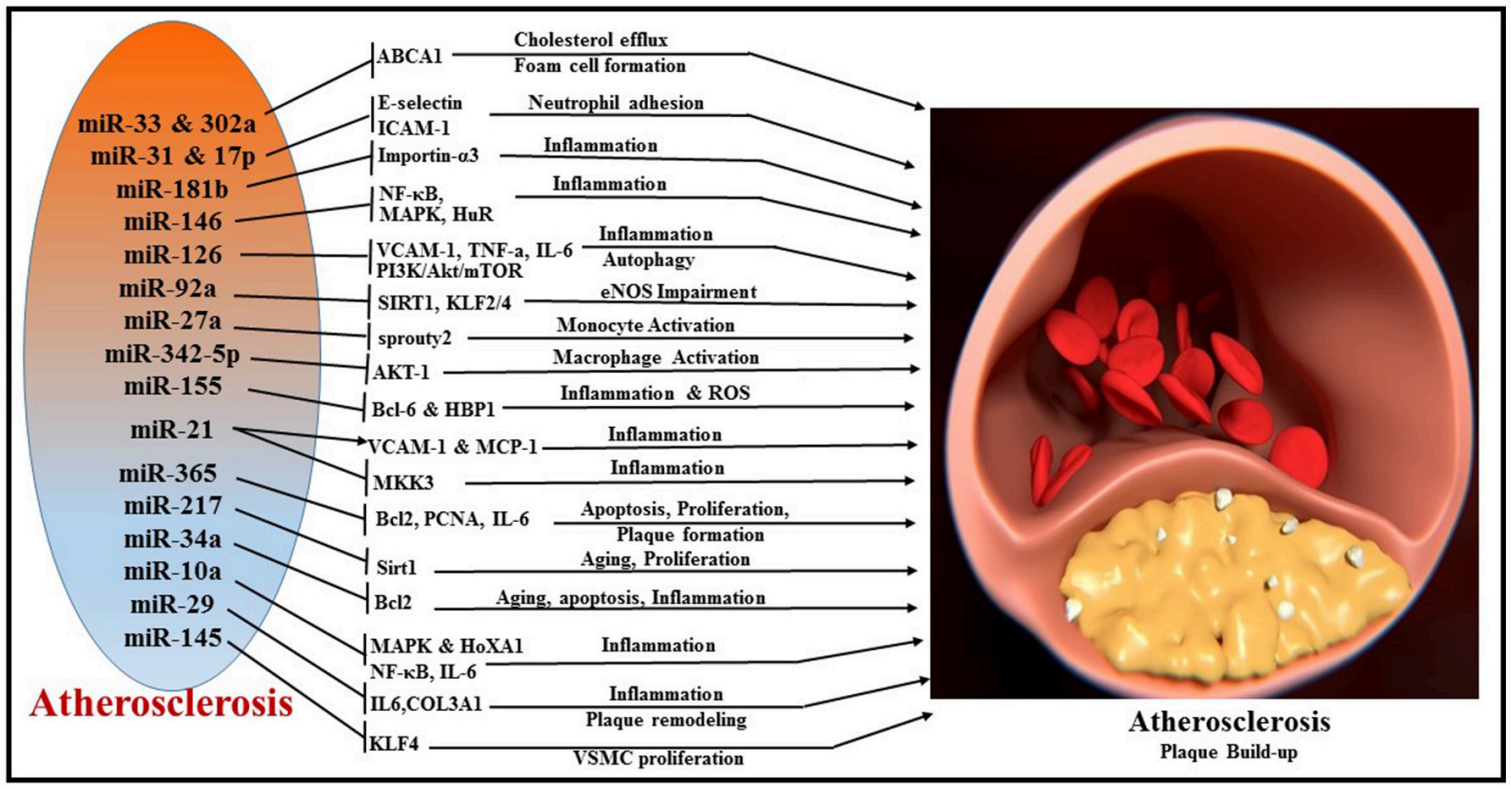
Overview of miRNAs and their respective target genes regulating multiple cellular processes, like cholesterol efflux, foam cell formation, neutrophil adhesion, inflammation, autophagy, monocyte activation, macrophage activation, apoptosis, cellular proliferation, and plaque remodeling during the pathogenesis of atherosclerosis.

Atherosclerosis is a progressive disease of the coronary arteries caused by plaque formation and lipid accumulation, accompanied by inflammation in the interior wall of blood vessels (149). The narrowing of the artery can limit or block coronary blood flow and lead to MI and related complications. miRNAs are important regulators of pathophysiological processes involved in the development of atherosclerosis such as cellular adhesion, proliferation, lipid uptake, and efflux, and recruitment of inflammatory mediators (Figure 6). The liver plays a central role in lipoprotein metabolism and several hepatic-enriched miRNAs have been identified to have significant impact on lipid homeostasis (150). Among these, hepatic-miR-122 was the first to be identified as a crucial regulator of cholesterol and fatty acid synthesis, and thus lipoprotein homeostasis (151, 152). A number of other miRNAs have been implicated in cholesterol efflux to apoA1, including miR-33 (153–155), miR-758 (156), miR-26 (157), miR-106 (158), and miR-144 (159). miR-33 is one of the most extensively investigated miRNAs and it represses multiple genes involved in cellular cholesterol trafficking (150). MiR-33a/b is embedded within introns of the SREBP (sterol regulatory element-binding protein) genes, the key transcription regulators of many cholesterogenic and lipogenic genes (160). In concert with the transcription of SREBP, miR-33 inhibits cellular cholesterol efflux by targeting ATP-binding cassette transporter A1 (ABCA1) and ABCG1 genes (161). Studies using ApoE/miR-33 double knockout mice demonstrated reduced atherosclerotic plaque with significant increase in HDL levels and enhanced cholesterol efflux via ABCA1 and ABCG1 (162). Interestingly, antagonism of miR-33 in Ldlr^−/−^ mouse models of atherosclerosis impeded the progression of atherosclerosis (163) and also regressed established atherosclerosis (155). Anti-atherosclerotic effects of anti-miR-33 therapy have been attributed to increasing circulating levels of HDL or improving macrophage cholesterol efflux via ABCA1 and ABCG1, two of the well-established targets of miR-33 (155, 162, 164). Macrophage-specific loss of miR-33 was determined to impede atherosclerotic plaque formation by reducing inflammation and lipid accumulation in Ldlr^−/−^ mice under hyperlipidemic conditions (165). Alternatively, whole body loss of miR-33 in Ldlr^−/−^ mice resulted in increased body weight, impaired insulin sensitivity, and a pro-atherogenic lipid profile without significant changes in the plaque size. Humans possess another isoform of miR-33, namely miR-33b. However, bone marrow transplants from miR-33b-KI mice into the Ldlr^−/−^ background did not show any impact on plaque formation or lipid accumulation (165). Recently, miR-302a and miR-26 have also been reported to be involved in cholesterol transport and efflux by targeting ABCA1 (157, 166).

Inflammatory activation of ECs promotes atherosclerosis by recruitment of leukocytes (167). Vascular adhesion molecule (VCAM)-1, intracellular adhesion molecule (ICAM)-1, and E-selectin are important players in the leukocyte recruitment to the vessel wall, which eventually leads to plaque formation (168). In human ECs, E-selectin and ICAM-1 are direct targets of pro-inflammatory cytokine TNFα-induced miR-31 and miR-17-3p, respectively, regulating neutrophil adhesion to ECs (169). MiR-181b regulates NF-κB-mediated EC activation, vascular inflammation as well as atherosclerosis via repression of importin-α3, a protein that is required for nuclear translocation of NF-κB (170, 171). MiR-146 maintains vascular homeostasis with repression of the pro-inflammatory signaling pathways, i.e., NF-κB pathway as well as the MAP kinase pathway and downstream early growth response (EGR) transcription factors through regulation of IL-1β signaling pathway adaptor proteins (i.e., TRAF6, IRAK1/2) (172). In addition, miR-146 modulates post-transcriptional pro-inflammatory pathways via targeting the RNA binding protein HuR (human antigen R), which promotes endothelial activation by antagonizing eNOS (endothelial nitric oxide synthase) expression (172).

A wealth of evidence suggests the involvement of miR-126 in the inflammatory responses associated with atherosclerosis (173, 174). MiR-126 suppresses VCAM-1 expression and limits leukocyte adherence to ECs and regulates vascular inflammation (138). Overexpression of miR-126 poses beneficial effects by decreasing the pro-inflammatory cytokine expression (TNF-α, IL-6) and reducing the accumulation of macrophages in atherosclerotic lesions by inhibiting MAPK pathway proteins (p38, ERK1/2, and JNK) (174). Another interesting study showed that overexpression of miR-126 prevented against ox-LDL-induced injury in HUVECs through restoring autophagy flux via repressing PI3K/Akt/mTOR pathway. This observation was supported by decreased LC3-II, Beclin 1, and p62 expression profiles that were induced by ox-LDL.

HUVECs treated with ox-LDL exhibited a robust increase in the expression level of miR-365 and downregulation of its target Bcl-2 (175). However, inhibition of miR-365 attenuated ox-LDL-induced EC apoptosis by restoring the expression of Bcl-2 (175). In contrast to this finding, miR-365 levels were downregulated in plaques (vs. healthy adjacent tissue) and in monocytes of coronary atherosclerosis (AS) patients compared to control subjects (176). Interestingly, the levels of IL-6 (direct target of miR-365), in both plaques and monocytes correlated with the expression level of miR-365 (176), suggesting a role for miR-365 in the pathogenesis of AS. MiR-365 was also reported to reduce proliferation of vascular smooth muscle cells (VSMCs) by targeting cell cycle-specific cyclin D1 (CD-1) both *in vitro* and in balloon injury-induced carotid artery proliferation model in rat (177). In specific, transfection of miR-365 mimics in VSMCs blunted PDGF (platelet-derived growth factor) or ANG-II-induced cell proliferation by decreasing the level of Proliferating Cell Nuclear Antigen (PCNA) through targeting CD-1 (177).

Recent studies reported that miR-92a is induced by oxidative stress in ECs (178) and is also involved in the development of atherosclerosis (179). MiR-92a targets the 3′ untranslated region of mRNAs encoding sirtuin 1 (SIRT1), Krüppel-like factor 2 (KLF2), and KLF4, and impairs eNOS-driven NO bioavailability (180). Specific *in vivo* inhibition of miR-92a expression in mice was shown to reduce endothelial inflammation and limit the development of atherosclerosis (179).

Macrophage foam cell formation, a hallmark of atherosclerosis, was determined to be regulated by miR-27a, which can activate CD14, CD68 expression, and CD206 and DC-SIGN, a marker of M2 and secretion of IL-10. Experiments using monocytes indicated that overexpression of miR-27a increased IL-10 secretion by activating ERK signaling pathway (181). MicroRNA expression profile reveals that macrophage-derived miR-342-5p and miR-155 are selectively upregulated in early atherosclerotic lesions in ApoE-knockout mice (182). This study indicates that miR-342-5p promotes atherosclerosis and induces the pro-inflammatory activation of macrophages by suppressing Akt1-mediated inhibition of miR-155 expression. In turn, miR-155 also promotes atherosclerosis by directly repressing the expression of BCL6 (B-cell leukemia/lymphoma 6), a transcription factor that attenuates pro-inflammatory NF-κB signaling (183). Systemic delivery of antagomiR-155 diminishes lipid-loading in macrophages and reduces atherosclerotic plaques in ApoE knockout mice (184). Ectopic overexpression and knockdown of miR-155 identified that HMG box-transcription protein 1 (HBP1) is a novel target of miR-155. miR-155, by direct repression of HBP1 expression, promoted lipid uptake and ROS production of macrophages to enhance foam cell formation (184). Furthermore, miR-155 also directly inhibits SOCS1 expression and induces p-STAT3 and PDCD4, which leads to production of inflammation mediators in macrophages to promote atherosclerotic plaque formation (185).

Aging is one of the major risk factors for type 2 diabetes mellitus and its associated endothelial dysfunction and atherogenesis (186). Interestingly, endothelial senescence seems to be dependent on the age-progressive increase in miR-217 (187). Upregulation of miR-217 was shown to negatively regulate Silent information regulator 1 (Sirt1) in human atherosclerotic plaques (187). This study reported a fascinating role linking miR-217 to aging ECs. Data showed a progressive upregulation of miR-217 during aging in cell lines including young human umbilical vein endothelial cells (HUVECs), human aortic ECs and human coronary artery ECs (187). Sirt1, a master regulator of aging was identified as a direct target of miR-217 and was shown to decline over age in correlation with potentiation of miR-217. Conversely, inhibition of miR-217 restored the levels of Sirt1 and modulated forkhead box O1 (FoxO1) and enhanced angiogenesis (187). Similarly, increased miR-34a in concert with suppression of SIRT1 expression were also reported in aged mouse aortas and in replicative senescent human aortic smooth muscle cells (HASMCs) (188). In addition, overexpression of miR-34a increased several pro-inflammatory factors like IL1β, IL8, IL6, and Mcp-1 in both endothelial and VSMCs (188). miR-34a was also found to be upregulated in HFD-induced ApoE^−/−^ mice and ox-LDL-treated HAECs (189). Inhibition of miR-34a decreased atherosclerotic lesions and reduced EC apoptosis in HFD-induced ApoE^−/−^ mice through suppression of its target Bcl-2 (189). Moreover, anti-miR-34a released the cell cycle arrest at the G1 phase induced by ox-LDL treatment of HAECs, suggesting that miR-34a promoted cell proliferation (189). Taken together, these reported findings imply that miR-34a regulates growth and apoptosis in ECs and plays an important role in atherosclerosis.

MiR-21 has received significant attention with respect to its role in CVDs because it was shown to be up-regulated in the arteries of patients with atherosclerosis (190). In early stages of atherosclerosis, miR-21 exhibits pro-inflammatory effect in ECs via activation of pro-inflammatory protein VCAM-1 and MCP-1 (monocyte chemotactic protein-1 (191). However, in later stages of the pathological process, it can facilitate suppression of inflammation via induction of eNOS with enhanced production of athero-protective NO, which suppresses activation and adhesion of monocytes and expression of pro-inflammatory cytokines (192). To this end, numerous studies identified the controversial role of miR-21, as a pro- or anti-atherogenic miRNA (193–195). A recent study revealed that miR-21 expression increased in macrophages and decreased in serum of patients with non-calcified coronary plaque. miR-21 participates in plaque instability by inducing the expression and secretion of pro-MMP-9 and active-MMP-9 in human macrophages via targeting gene RECK (Reversion-inducing cysteine-rich protein with Kazal motifs) (196). A report also suggested that miR-21 is the most abundantly expressed miR in macrophages and its absence leads to atherosclerosis in Ldlr^−/−^ mice fed with western diet (197). Initial data from RNA sequencing using bone marrow-derived macrophages (BMDMs) identified rich expression of miR-21 in macrophages (197). Further experiments showed that *Ldlr*^−/−^ mice that received BM from miR-21-deficient mice developed larger lesions than mice transplanted with wild type BM. In this context, mitogen-activated protein kinase kinase 3 (MKK3), a target gene of miR-21, was significantly increased in macrophages derived from miR-21^−/−^ mice, which resulted in the activation of the p38 MAP Kinase-C/EBP homologous protein (p38-CHOP) and c-Jun N-terminal kinase (JNK) signaling pathways (197). The study also revealed that the absence of miR-21 reduced expression of the ATP-binding cassette transporter G1 (ABCG1), thus promoting the development of foam cell formation (197).

Shear stress plays an important role in the induction of inflammation in ECs and contributes to the severity of atherosclerosis by increasing proinflammatory factors in plaque regions (198–200). In this regard, miR-663 was reported to regulate shear stress in ECs (201). miRNA microarray analysis using HUVECs identified an upregulation of miR-663 upon exposure to oscillatory shear stress (OS) for 24 h (201). Antagonism of miR-663 using miR-663-locked nucleic acids (LNAs) blocked OS and TNF-α induced monocyte adhesion (201). This study identified 35 potential miR-663 targets including inflammatory genes (BMP, IL-6, and PCK) and transcription factors (FOSB, CEBPB, DDIT3, ATF3, and MYCN) that are differentially regulated with OS HUVECs (201). These observations suggest that miRNAs, including miR-663, are sensitive to shear stress and can play a delicate role in regulating inflammation and plaque formation during atherosclerosis. Similarly, studies using inner aortic arch of pig suggest that miR-10a expression was significantly reduced in the athero-susceptible regions (202). Overexpression of miR-10a inhibited the expression of VCAM-1 and E-selectin as well as phosphorylation of IκBα and NF-κB signaling in human aortic ECs. Experiments using knock-in and knockdown of miR-10a suggested that miR-10a regulates proinflammatory endothelial phenotypes in athero-susceptible regions both *in vivo* and in ECs by targeting NF-κB, MAPK and Homeobox A1 (HOXA1) genes (202). Importantly, a recent study reported lower expression of miR-10a and simultaneous higher expression of IL-6 and TNF-α in peripheral blood mononuclear cells (PBMCs) of patients with coronary artery disease (CAD) compared to control subjects (203).

Apart from mechanical shear stress, abnormal remodeling of plaques also increases its susceptibility to rupture. To this end, miR-29 was shown to impose positive remodeling of plaque and thus reducing the risk of plaque lesion (204). Interestingly, administration of LNA-miR-29 biweekly for 14 weeks reduced atherosclerotic lesion size in APOE^−/−^ mice fed with high fat diet (204). Further, LNA-miR-29 increased fibrous cap thickness and SMA staining and reduced necrotic zones in lesions. Mechanistically, LNA-miR-29 increased collagen COL1A and COL3A1 (targets of miR-29) only in the risk-prone plaque region and stabilized them without inducing systemic fibrosis (204). Similarly, overexpression of miR-145 in APOE^−/−^ mice before the onset of western diet for 12 weeks displayed reduced plaque formation (205). Specifically, VSMC-targeted expression of miR-145 resulted in plaque stability and decreased macrophage infiltration (205). Furthermore, overexpression of miR-145 resulted in a reduction in KLF4 levels with a concomitant increase in myocardin expression to promote a contractile phenotype of VSMC (205).

### MicroRNAs in diabetes and insulin signaling

**Figure 7:**
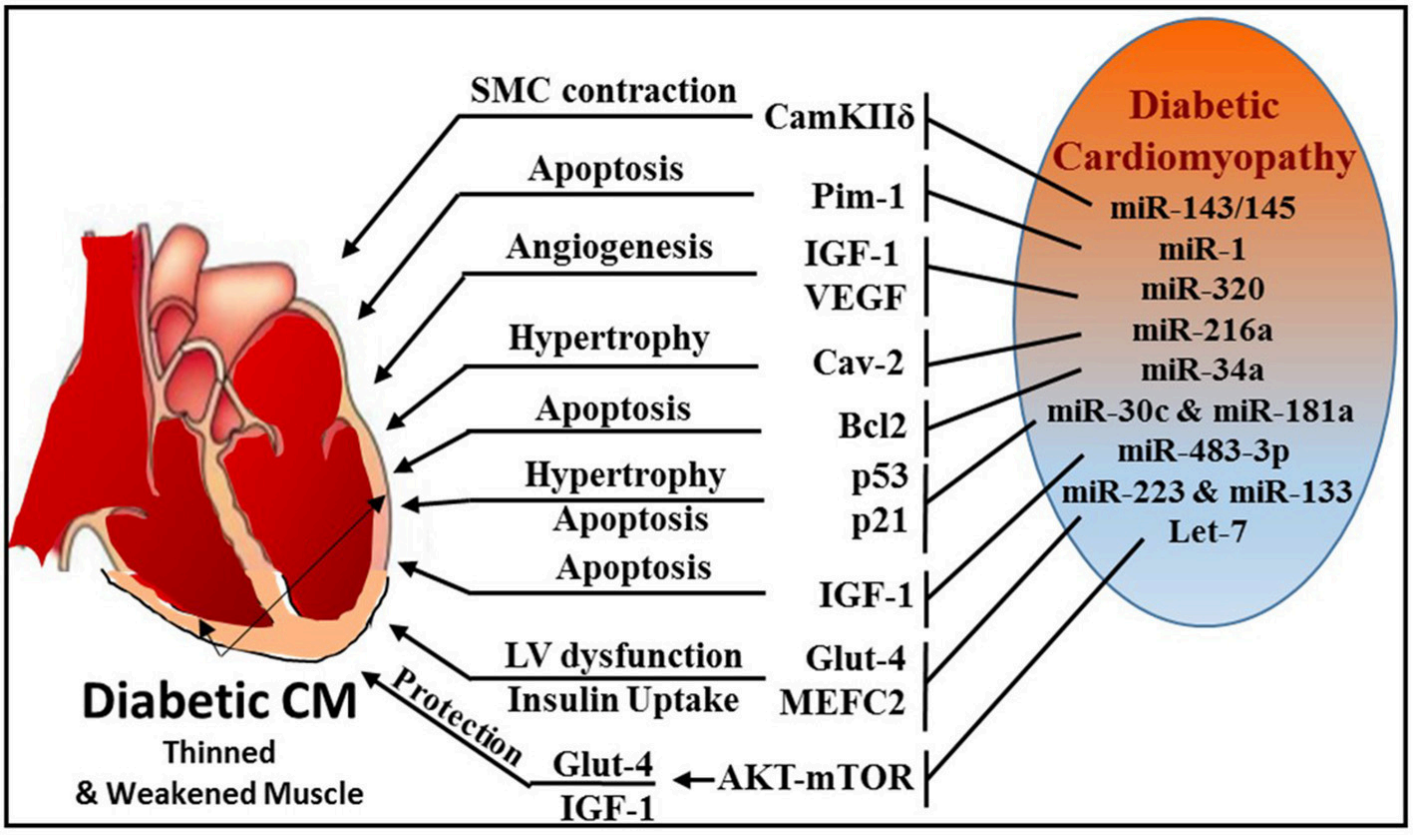
Schematic diagram illustrating multiple miRNAs and their targets, which mediate various cellular events, like SMC contraction, apoptosis, angiogenesis, hypertrophy, and glucose homeostasis during diabetic cardiomyopathy.

Diabetes is a major risk factor for CVD and is characterized by elevated blood glucose, insulin resistance/deficiency and metabolic abnormalities. Several miRNAs were identified to play a role in diabetes by regulating insulin signaling and glucose metabolism (Figure 7). Some of the prominent players are miR-34a (206), miR-204 (207), miR-103/107 (208), miR-134 (209), miR-130a (210), miR-155 (211), miR-21 (212), miR-320 (213), and miR-27b (214).

Formation of Advanced Glycation Products (AGE) that leads excessive reactive oxygen species (ROS) generation is a major mechanism of diabetes-related complications (215, 216). AGE plays an important role in the activation of PKC/Rho pathway induced by hyperglycemia (217, 218). Using Akita type 1 diabetic mice and miR-143/145 cluster knockout mice, Hien et al. established a pivotal link between hyperglycemia and smooth muscle cell (SMC) contractility (219). This study demonstrated that inhibition of PKC/Rho/MRTF (myosin phosphatase-targeting protein) signaling and genetic knockdown of miR-143/145 cluster reduced glucose-induced contractile gene expression.

Zampetaki et al. revealed that reduced miR-126 expression levels are responsible for impaired vascular repair capacities in diabetes (220). Patients with type 2 diabetes exhibited significant reduction in the level of vesicular miR-126, while non-vesicle-associated miR-126 was unchanged. Jansen et al. also reported decreased miR-126 in circulating micro-particles from patients with stable CAD and diabetes (221).

miR-1 was also found to play an important role in diabetes by directly targeting insulin-like growth factor-1 and its receptor (222) or signaling cascades related to IGF pathway (223). MiR-1 increases from the early to the late phases of diabetic cardiomyopathy, which leads to consequential cardiomyocyte apoptosis through targeting Pim-1 (proviral integration site for Moloney murine leukemia virus-1) (224). Intriguingly, blocking miR-1-dependent inhibition of Pim-1 using anti-miR-1 resulted in elevation of phosphorylated Akt and abrogation of diabetic-induced cardiac apoptosis.

Wang et al. reported that miR-320 was directly involved in regulation of insulin-like growth factor-1 in T2D rats and led to impaired angiogenesis in diabetes (213). The study also revealed that in diabetic myocardial microvascular endothelial cells (MMVECs), miR-320 is potentially targeting multiple angiogenesis-related genes, including Flk-1 (fetal liver kinase), VEGF, IGF-1 (insulin growth factor-1), IGF-1R (IGF-1 receptor), and FGFs (fibroblast growth factors). A notable finding was reported by Trajkovski et al. showing upregulation of miR-103/107 in obese mice. Adenovirus-mediated overexpression of miR-103/107 impaired glucose homeostasis in liver and fat, whereas antagomir-based inhibition of miR-103/107 increased insulin sensitivity and glucose uptake. The study also implicated caveolin-1 as a functional target gene of miR-103/107. Whether such mechanism exists in diabetes-related cardiovascular complications remains to be answered. Another miR, namely miR-216a, also targets caveolin-2, a scaffolding protein and substrate of the insulin receptor that helps recruit IRS-1 to the insulin receptor and propagate insulin signaling (225). Loss of caveolin expression results in the activation of a program of progressive hypertrophy in cardiac myocytes, and its deletion results in severe cardiomyopathy (226). Greco et al. revealed that miR-216a is induced in failing hearts of patients with and without diabetes, and this induction is negatively correlated with left ventricular ejection fraction (225).

The expression of miR-34a was found to be highly induced in H9c2 cells subjected to high glucose and concurrently, Bcl-2 expression was markedly reduced (227). *In vivo* experiments using miR-34a mimic prevented cardiac recovery post-MI in neonatal hearts whereas inhibition of miR-34a in adult hearts improved cardiac repair (228). Many key proteins involved in apoptosis and necrosis, such as Bcl-2, CD-1, and SIRT1 are also regulated by miR-34a (228). He et al. identified that members of the miR-34 family are direct transcriptional targets of p53 (229) and p53 is stimulated in high glucose cultured cardiomyocytes with induction of miR-34, which was also associated with a marked diminution of pro-survival SIRT1 (230). Therefore, the induction of miR-34 in diabetic heart may promote cardiomyocyte apoptosis in diabetic patients. Furthermore, miR-30 and miR-181a have also been recently shown to regulate p53 expression in cardiomyocytes (231, 232). Raut et al. revealed that myocardial expression of p53 and p21 genes is increased with simultaneous reduction in miR-30c and miR-181a in hearts of diabetic patients, rats with diabetic cardiomyopathy and in high glucose-treated cardiomyocytes (233).

MiR-483-3p is a critical regulator of heart development and a prognostic factor for heart disease (234). Induction of diabetes using streptozotocin in miR-483 transgenic mouse model increased cardiomyocyte apoptosis by silencing insulin growth factor 1 (IGF-1) (235). IGF-1 is known regulator of Myocyte enhance factor 2C (MEF2C), which plays an active role in diabetes-provoked cardiac hypertrophy (236). In this regard, it was observed that downregulation of miR-133a in diabetic cardiomyopathy resulted in an upregulation of serum and glucocorticoid regulated kinase 1 (SGK1) and IGFR1 (237). This in turn leads to the activation of MEF2C and p300 gene, paving the way for myocyte hypertrophy. On the contrary, miR-133 was also shown to target MEF2C and suppress it expression thereby blocking hypertrophy (237, 238). Downregulation of miR-133a is associated with induction of cardiac autophagy in diabetic patients with HF (177). MiR-133a improves the contractility of diabetic hearts by targeting tyrosine aminotransferase which leads to induction of norepinephrine biosynthesis, and consequently, activating β-adrenergic receptor (175).

Glucose transporter 4 (Glut4) is a major regulator that facilitates entry of glucose into cells and miR-223 was reported to control the expression of Glut4 gene in cardiomyocytes. A broad miRNA analysis study using left ventricular biopsies from patients with or without type 2 diabetes who presented with left ventricular dysfunction revealed that miR-223 was robustly upregulated in patient with diabetes, whereas Glut4 expression was low (239). Moreover, Horie et al. found that miR-133 overexpression lowered Glut4 levels by targeting KLF15 (Kruppel-like factor) and reduced insulin-induced glucose uptake in cardiomyocytes (240).

Overexpression of let-7 has been reported to mediate insulin resistance and impair glucose metabolism in high fat diet-induced diabetic mice (241), whereas the inhibition of let-7 resulted in improvement of glucose metabolism and insulin sensitivity (242). Recently Li et al. established that inhibition of the let-7 family improves glucose uptake and insulin resistance in streptozotocin-induced diabetic rats and confers cardioprotection against I/R injury through Akt and mTOR pathways (243). The study also determined that inhibition of let-7 enhanced the expression of IGF-1R, InsR (insulin receptor), and Glut4 in diabetic hearts.

Zheng et al. demonstrated that miR-195 is induced in streptozotocin-induced type 1 and db/db type 2 diabetic mouse hearts with reduction of its target proteins (B cell leukemia/lymphoma 2, Bcl-2 and sirtuin 1, Sirt1) (244). They also indicated that upregulation of miR-195 in diabetic hearts is associated with oxidative stress, apoptosis, myocardial hypertrophy, and dysfunction as well as reduction in coronary blood flow. Additionally, silencing of miR-195 reduced myocardial hypertrophy and apoptosis, increased myocardial capillary density and improved coronary blood flow and myocardial function in diabetic mice.

## ncRNA as predictors and prognostic tools in CVD

The identification of circulating miRNAs in blood and other liquid samples has immediately gained the attention of clinical research for their potential utility as biomarkers. Perhaps one of the most highly focused areas of research in ncRNAs is miRNAs as predictors and prognostic tools for human diseases (Table 1). These miRNAs are quite stable and withstand degradation in the blood stream largely due their association with proteins (266), apoptotic bodies (267), microvesicles (268), and exosomes (269). The feasibility of obtaining serum samples from human patients accelerated the research in this field and therefore several miRNAs were identified as biomarkers for various diseases such as cancer (270, 271), diabetes (272, 273), and CVD (274, 275).

**Table 1:** Non-coding RNAs as biomarkers in cardiovascular diseases.

**ncRNA**	**Disease**	**Expression**	**Model**	**Type of marker**	**References**
miR-1	AMI	Up-regulated	Rat, pig (CAO) Human (TASH, STEMI, ACS)	Diagnostic	(245–250)
miR-133a and b	AMI	Up-regulated	Pig (CAO) Human (AMI, ACS)	Diagnostic	(247–249, 251)
miR-499	AMI HF	Up-regulated	Human (STEMI)	Diagnostic/Prognostic	(247, 250)
miR-134, miR-198	CAD	Up-regulated	Human		(252)
miR-208b, miR-499-5p	MI HF	Up-regulated	Human (STEMI, ACS)	Prognostic	(245, 248–250, 252, 253)
miR-126,	Ischemic HF CAD, T2DM	Down-regulated	Human	Prognostic	(253–255)
miR-508-5p	HF	Up-regulated	Human	Prognostic	(253)
miR-18a-5p and miR-652-3p	Acute HF	Down-regulated	Human (AHF, COPD)	Predictor	(256)
miR-483-3p	LVAD	Up-regulated		Diagnostic/Predictor	(256–258)
miRNA-26b-5p, miRNA-145-5p, miR-1202	Heart Failure LVAD	Up-regulated	Human (CRT)	Predictor/Prognosis	(257, 258)
miR-28-3p	T2DM	UP-regulated	Human	Diagnostic	(220)
miR-20b, miR-21, miR-24, miR-191	T2DM, CAD	Down-regulated	Human	Diagnostic	(220)
miR-663b	AMI	Up-regulated	Human	Diagnostic	(259)
miR-181	IR injury	Up-Regulated	Rat, Pig	Diagnostic	(260)
miR-30a	AMI	Up-regulated	Human	Diagnostic	(261)
LIPCAR	AMI,	Down-regulated	Human	Diagnostic	(262)
NRON, MHRT	HF	Up-regulated	Human	Diagnostic	(263)
piR_2106027	MI	Up-regulated	Human	Diagnostic	(264)
Circ-MICRA	AMI	Down-regulated	Human	Prognostic	(265)

*CAO, Coronary Artery Occlusion; CAD, Coronary Artery Disease; AMI, Acute Myocardial Infarction; HF, Heart Failure; MI, Myocardial Infarction; TASH, Trans-coronary Ablation of Septum Hypertrophy; STEMI, ST-elevation myocardial infarction; ACS, Acute coronary syndrome; AHF, Acute Heart Failure; COPD, Chronic obstructive pulmonary disease; LVAD, Left Ventricular Assist Device*.

In patients with acute MI, miR-1, miR-133a, miR-499, and miR-208a have consistently been reported to be elevated in plasma (245, 246, 276). Clinicians are in pursuit of a reliable miRNA marker, similar to cardiac troponin, to evaluate the extent of MI injury. Numerous clinical studies indicated that miR-1 is markedly increased in the blood of patients with acute MI (245–247, 276). Similar to miR-1, miR-133 was also increased in plasma after coronary artery ligation in rats (245). Interestingly. miR-133 was also elevated in plasma of acute MI patients and positively correlated with cardiac troponin levels (251). A recent study conducted in patients with hypertrophic obstructive cardiomyopathy undergoing trans-coronary ablation of septal hypertrophy (TASH) procedure to identify time-dependent release of acute miRNAs that may be specific to cardiac tissue as an indicator of cardiomyocyte necrosis. The study showed that miR-1, miR-133a, and miR-208a increased continuously during the first 4 h post-MI (248). Interestingly the plasma concentration of miR-1 significantly increased (>3-fold) as early as 15 min after MI and reached peak level (>60-fold) after 75 min. A similar trend was also observed for miR-133a. In line with this finding, a comparative study between human and murine circulating miRNAs determined that the concentration human of miR-1, miR-133a, and miR-133b peaked even before cardiac troponin T post-MI, whereas in mice undergoing permanent coronary artery occlusion, miR-499 appeared to be a more sensitive marker of acute MI (247). Besides serving as diagnostic markers, miRs were also identified as predictors of disease prognosis. In a large cohort study involving 444 patients with acute coronary syndrome (ACS), the expression of miR-1, miR-133a, miR-133b, and miR-208b, were higher compared to patients with unstable angina (249). These patients were monitored for 6 months and the study concluded that miR-133a and miR-208b levels were significantly associated with the risk of mortality (249). In another interesting study involving 424 patients suspected for MI during a 30-day follow-up period, elevated plasma levels of miR-208b and miR-499-5p were strongly associated with increased risk of mortality or HF (250). Another study also suggested that the high expression level of a cluster of three miRs, including miR-134, miR-198, and miR-370, can also be used to distinguish between with CAD patients and healthy subjects (252).

Several reports suggest that miRNA expression can indicate the response to therapy. In this regard, miR-126 and miR-508-5p served as independent prognostic factors of chronic HF secondary to ischemic cardiomyopathy or non-ischemic cardiomyopathy (253). Recently, a study in patients with acute HF revealed that declining levels of circulating miR-18a-5p and miR-652-3p are associated with increasing acuity of HF (256). Interestingly, miRNAs may also serve as effective biomarkers in distinguishing responders and non-responders who received left ventricular assist device (LVAD) and cardiac resynchronization therapy (CRT) (257, 258) procedures. Patients who received LVAD had a consistently high expression of miR-483-3p, whereas levels of miR-1202 were able to identify responders vs. non-responders. The study also showed that patients with higher expression of miRNA-26b-5p, miRNA-145-5p, miRNA-92a-3p, miRNA-30e-5p, and miRNA-29a-3p responded well to CRT (257, 258).

Several studies have been conducted to examine whether the levels of circulating miRs can assist in outcome prediction of diabetic patients with impaired glucose metabolism and high-risk cardiovascular complications (220, 221, 254). A cohort study involving 80 patients with type 2 diabetes showed upregulation of miR-28-3p and downregulation of miR-20b, miR-21, miR-24, miR-15a, miR-126, miR-191, miR-197, miR-223, miR-320, and miR-486 (220). The study also used Lep(ob) mice to show a decline in miR-126 content of endothelial apoptotic bodies upon exposure to high glucose concentrations. Recently, it was demonstrated that expression levels of circulating miR-126 were decreased in the blood samples of type 2 diabetic patients with or without CAD (254). It has also been suggested that miR-126 correlates negatively with LDL in diabetic patients with CAD (254, 255). Circulating levels of miR-663b, was shown as a reliable marker for atherosclerosis related acute myocardial infarction (AMI) (259).

### MicroRNAs and exosomes

Recently researchers have shown interest in miRNAs packed in exosomes, largely due to their role in cell-cell communication (277) and ease of delivery into cells. Exosomes are small transport vehicles that measures 40–100 nm in diameter and are secreted membrane vesicles that originate from intracellular endosomes (278–280). Exosome-mediated cellular communication has been shown to play an important role in MI (281, 282). Transport of miRNA via exosomes can act as a potential mechanism for molecular cross-talk of combined gene and cell therapy in ischemic heart disease (283). Ibrahim et al. established that cardiosphere-derived cell exosomes (CDC_exo_) contain a distinctive complement of miRNAs, with particular enrichment of miR-146a (284). MiR-146 in exosomes plays a key role in mediating the beneficial effects of CDC_exo_ in infarcted heart, but alone does not suffice to confer comprehensive therapeutic benefit. Circulating levels of miR-663b were shown to serve as a reliable marker for atherosclerosis related to AMI (259).

Recently de Couto et al. proposed that CDC_exo_ contains several miRNAs, including miR-146a, miR-181b, and miR-126 and when delivered at reperfusion limits infarct size in a pig model of MI (260). They also showed that miR-181b within CDC_exo_ is a critical mediator of macrophage polarization *in vitro* and cardioprotection *in vivo*. Using EC-specific miR-126 knockout mice, Chen et al. showed a brain-to-heart communication through miR-126 in cerebral artery occlusion model (285). Interestingly, studies conducted in rat showed that miR-17-92 cluster enriched exosome delivery restores function after stroke via targeting PTEN-PI3K pathway (286).

Yang et al. detected that miR-30a is highly enriched in exosomes from the serum of acute MI patients *in vivo* and also in culture medium of cardiomyocytes after hypoxic stimulation *in vitro* (261). The study showed that hypoxia inducible factor (HIF)-1α controls miR-30a, which is efficiently transferred via exosomes between cardiomyocytes after hypoxia. Exosomes released from hypoxic cardiomyocytes inhibit autophagy by transferring miR-30a in a paracrine manner.

In addition, specific lipids found on lipoproteins, such as phosphatidylcholine (PC), have been shown to form stable ternary complexes with RNAs (287). In addition to exosomes, lipoproteins—especially high-density lipoprotein (HDL)—play a critical role as carriers of miRNAs in cardiometabolic disorders (288). HDL transports endogenous miRNAs and delivers them to recipient cells with functional gene regulatory consequences (289). Cellular export of miRNAs to HDL is regulated by neutral sphingomyelinase. Moreover, mouse models of hypercholesterolemia and dyslipidemia exhibit a significantly distinct HDL-miRNA profile compared to healthy subjects, indicating that miRNA cargo of HDL may be involved in the atherosclerotic disease and cardiometabolic disorders (289). HDL-bound miRNAs may also be used as biomarkers in cardiometabolic disorders (290). Several angiogenesis and inflammation-associated miRNAs, including miR-92a, miR-126, miR-150, miR-378, and miR-486 were also found in circulating HDL of patients with CAD (288, 291). A recent study revealed that normalization of miRNAs with HDL level shows significant decrease in cardio-enriched miRNAs (particularly miR-1, 133, and 499) in diabetic patients undergoing coronary artery bypass graft (CABG) surgery for ischemic heart disease (292). The study proposed the need to normalize miRNA levels with HDL to increase its sensitivity as a diagnostic biomarker.

Overall, accumulating evidence implicates multiple circulating miRNAs as potential diagnostic tools as well as prognostic biomarkers of CVD. Therefore, multicenter and large cohort studies should be carefully designed to further identify and confirm specific circulating miRNAs as novel biomarkers for early diagnosis and/or prognosis of CVD in patients.

## LncRNA in cardiovascular diseases

LncRNAs regulate various biological processes, including cell proliferation, differentiation and apoptosis (293, 294), and are aberrantly expressed in several pathological conditions such as CVD (2), diabetes mellitus (5, 295), and cancer (296, 297). Moreover, the expression of lncRNAs is predominantly unique to tissues and cell types (49, 297) and lncRNAs are therefore relatively precise in their functionality. LncRNAs act as powerful epigenetic modulators and also play an important role in heart development. Global transcriptome analyses identified deregulation of thousands of novel lncRNAs during cardiac development and pathology, but only a few have been well-characterized (298–302) (Table 2). Perhaps, one of the earliest known lncRNA is myosin heavy chain-associated RNA transcript (Mhrt/myheart), which plays a role in cardiomyocyte proliferation (304). Mhrt is inhibited by Brg1-HDAC-PARP—a chromatin repressor, which governs the transition of alpha-MHC to beta-MHC. Pathological stress activates Brg1, represses Mhrt and results in cardiac hypertrophy in adults (304).

**Table 2:** Long non-coding RNAs and their function in the cardiovascular system.

**LncRNA**	**Disease**	**Expression**	**Functional outcome**	**Target regulation**	**References**
Braveheart	Cardiac hypertrophy	Down-regulated	Cardiac differentiation Cardiac lineage	Mesoderm posterior1 SUZ12, *Gata4*	(303)
Mhrt/myheart	Cardiac hypertrophy	Down-regulated	α-MHC–β-MHC	Brg1	(304)
Chaer	Cardiac hypertrophy	Up-regulated	Methylation of histone	PRC2, mTORC1	(302)
Chast	Cardiac hypertrophy	Up-regulated	Cardiomyocyte autophagy	Plekhm1	(305)
CHRF	Cardiac hypertrophy	Up-regulated	Sponge of miR-489	miR-489, Myd88	(306)
ROR	Cardiac hypertrophy	Up-regulated	Sponge of miR-133, fetal gene expression	miR-133, ANP and BNP	(307)
NRF	Myocardial infarction/IR injury	Up-regulated	Necrosis of cardiomyocytes	miR-873, RIPK1/RIPK3	(308)
APF	Ischemia reperfusion injury	Up-regulated	Suppress miR-188-3p	miR-188-3P, ATG7	(309)
CAIF	Myocardial infarction, Dilated cardiomyopathy	Up-regulated	Sarcomere development, autophagy	P53, LC3-II	(310, 311)
H19	Cardiac hypertrophy, Atherosclerosis	Down-regulated	VSMC proliferation and apoptosis	miR-675, CaMKIId, P38 and P65, MAPK and NF-kB	(312, 313)
MIAT	Myocardial infarction, hypertrophy	Up-regulated	Biomarker for MI	miR-150	(314, 315)
LIPCAR	Myocardial infarction	Up-regulated	Biomarker for MI and predictor of mortality	Mitochondrial Pathway	(262, 316)
MALAT1	Hind limb ischemia	Up-regulated	Angiogenesis	MMP-2	(317)
BACE1	Ischemic heart failure Atherosclerosis	Up-regulated	Biomarker for heart failure	Akt-mTOR pathway	(318, 319)
NRON and MHRT	Heart failure	Up-regulated	Biomarker for heart failure	Myosin Heavy Chain, HDL, LDH	(263)
CARL	Myocardial infarction	Up-regulated	Mitochondrial fission	miR-539, PHB2	(320)
Meg3	Myocardial hypertrophy	Up-regulated	Cardiac remodeling	MMP-2, p53	(321)
ANRIL	Atherosclerosis CAD	Up-regulated	Cell proliferation	CDKN2A/B	(322–325)
SENCR	Diabetes	Down-regulated	Biomarker for LV dysfunction in T2DM	FOXO1, TRPC6	(31, 262, 316)
SMILR	Atherosclerosis	Down-regulated	VSMC proliferation	HAS2	(326)
HOTAIR	AMI, hypertrophy	Down-regulated	Biomarker of MI, myocardial apoptosis,	miR-1, miR-19	(327, 328)
E330013P06	Atherosclerosis Diabetes	Up-regulated	Inflammation and macrophage activation	*Cd36*, inflammatory genes	(329)

Recent pioneering work has identified a significant role for Braveheart (BVHT) lncRNA in cardiac lineage (303). It was observed that Bvht directs mesoderm toward cardiac fate via mesoderm posterior 1 (MesP1), a mediator of cardiovascular progenitors. The study also showed that Bvht interacts with SUZ12, a core component of polycomb-repressive complex 2 (PRC2), suggesting an epigenetic regulation of chromatin. Moreover, several important transcription factors necessary for the commitment of cardiac lineage such as *MesP1, Gata4, Hand1, Hand2, Nkx2.5*, and *Tbx5* are activated by Bvht.

LncRNAs are also being recognized as rigorous regulators of DNA methylation through interactions with DNA methyltransferases (DNMTs) and thereby act as epigenetic modulators both in normal and in pathological conditions (330). Evidence also suggests that cardiac-enriched lncRNAs, such as Cardiac Hypertrophy-Associated Epigenetic Regulator (Chaer), can block the methylation of histone H3 lysine 27 by interacting with polycomb repressor complex 2 (PRC2) and contribute to cardiac hypertrophy (302). This Chaer-PRC2 interaction is transiently enhanced at the onset of hypertrophic stress in a mammalian target of rapamycin complex 1 (mTORC1)-dependent manner, and is prerequisite for epigenetic reprogramming and induction of hypertrophic genes. Both genetic and siRNA-mediated inactivation of Chaer significantly attenuate cardiac hypertrophy and pathological progression. Cardiac Hypertrophy-Associated Transcript (Chast) is another novel cardiomyocyte-specific lncRNA known to be upregulated in TAC-induced hypertrophy in mice and aortic stenosis patients (305). It was shown that Chast impedes the expression of Pleckstrin homology domain-containing protein family member 1 (Plekhm1) as a cis regulatory element, which hampers cardiomyocyte autophagy and promotes hypertrophy.

Interestingly, lncRNAs can also interact with miRNAs and act as a decoy to regulate gene expression. Cardiac Hypertrophy-Related Factor (CHRF), another lncRNA, is able to directly bind to miR-489 and regulate the expression of MyD88 (myeloid differentiation primary response gene 88, as a miR-489 target) and consequent cardiac hypertrophy (306). MyD88 knockout mice and transgenic miR-489 overexpressing mice are resistant to hypertrophic stimuli with AngII treatment. Mechanistically it was shown that CHRF binds to miR-489 and acts as an endogenous sponge of miR-489 to downregulate its expression. Another lncRNA, ROR, was identified to be upregulated in hypertrophic mouse heart and cardiomyocytes (307). The pro-hypertrophic effect of lncRNA-ROR is mediated via repressing the expression and function of miR-133, overexpression of which attenuates lncRNA-ROR and expression of fetal genes (ANP and BNP). As evident from recent findings, gene regulation and the interplay among networks of ncRNAs is increasingly complex and yet precise. One such example is a three-way interaction between lncRNA H19, miR-675, and CaMKIId that was established in a mouse model of phenylephrine-induced hypertrophy (312). The study showed that overexpression of H19 attenuates cardiomyocyte hypertrophy in response to phenylephrine, whereas knock-down of H19 exacerbates it. Furthermore, *in vivo* silencing of miR-675 in a pressure overload-induced mouse model of HF increases cardiac CaMKIIδ expression and aggravates cardiac hypertrophy. Moreover, H19 was shown to be elevated in serum of patients with atherosclerosis as well as in atherosclerotic plaques of ApoE-knockout mice treated with high-fat diet (313). The study also demonstrated that overexpression of H19 enhances the expression of P38 and P65 and increased proliferation while reducing apoptosis in VSMC and HUVECs). The data suggested that H19 may regulate MAPK and NF-kB in atherosclerosis. Human MI-associated transcript (MIAT) was identified as a novel lncRNA associated with increased risk of MI (314). Vausort et al, reported that MIAT is significantly elevated in whole blood from patients with non-STsegment-elevation MI (NSTEMI) compared to STEMI patients, suggesting that MIAT may be associated with chronic cardiomyopathy. A recent study also revealed that MIAT is significantly increased in Ang II-induced cardiac hypertrophy in mice and in H9c2 cells with reduction of miR-150 (315). Accordingly, the study suggested that MIAT acts as sponge to inhibit miR-150 expression and enhanced cardiac hypertrophy.

Similarly, another lncRNA, Necrosis-Related Factor (NRF) served as a sponge to reduce the expression of miR-873 (308). MiR-873 suppresses myocardial infarct size upon experimental I/R injury by reducing the translation of RIPK1/RIPK3 as well as RIPK1/RIPK3-mediated necrotic cell death in cardiomyocytes. Knockdown of NRF reduces necrosis and MI upon I/R injury. The transcription factor p53 has been identified as an activator of NRF expression and regulates cardiomyocyte necrosis and myocardial I/R injury through NRF-miR-873-RIPK1/RIPK3 pathway.

Autophagy is a clearance mechanism where degraded proteins or damaged organelles are removed from the system to alleviate cellular burden (331). A recent study revealed that among lncRNAs, only AK079427, named as autophagy promoting factor (APF) is significantly upregulated following I/R injury in mouse heart along with reduction in miR-188-3P (309). The study also shows that enforced expression of miR-188-3p *in vivo* attenuates autophagy and myocardial infarct size in response to I/R injury by targeting ATG7, an autophagy-regulating protein. Accordingly, the study indicates that I/R induces APF expression, which interacts with miR-188-3p and inhibits its repressing activity on its downstream target ATG7, and finally leads to autophagy. Similarly, cardiac autophagy inhibitory factor (CAIF), was reported to suppress cardiac autophagy and attenuate MI (310). CAIF directly interacts with p53 protein and prevents its binding to the promotor region of myocardin and abolishes its transcription. The loss of myocardin, in turn, decreases the accumulation of LC3-II and attenuates autophagy. However, reports also suggest that myocardin transcription factor in cardiomyocytes is required for healthy sarcomere development. Ablation of myocardin causes loss of cardiomyocytes due to increased apoptosis and results in dilated cardiomyopathy (311).

The homeostasis of mitochondrial dynamics in heart during I/R injury is critical. Mitochondrial fusion is able to inhibit apoptosis, while growing body of evidence indicates that abnormal mitochondrial fission could initiate cellular apoptosis in the pathogenesis of many diseases (320). Prohibitin complexes, PHB1 and PHB2, are present in the inner mitochondrial membrane and play an important role in mitochondrial fusion and fission (332). PHB1-overexpressing transgenic mice that are subjected to MI showed reduced mitochondrial fission and lesser myocardial infarct size (333). On the other hand, Wang et al. demonstrated that overexpression of PHB2 inhibits post-ischemic mitochondrial fission with reduction in myocardial apoptosis and MI (320). They also identified that PHB2 is negatively regulated by miR-539, which can affect mitochondrial fission and apoptosis. Intriguingly, the same study revealed that cardiac apoptosis-related lncRNA (CARL) acts as a sponge and negatively regulates miR-539 and enhances PHB2 expression to inhibit mitochondrial fission and myocardial apoptosis, consequently attenuating MI. Another mitochondrial lncRNA, LIPCAR/uc022bqs.1 (long intergenic non-coding RNA predicting cardiac remodeling), was found to be decreased early during MI, but upregulated during later stages. Circulating LIPCAR was used as a biomarker for MI and as a prognostic tool for cardiac remodeling (262, 316).

In a landmark study, Michalik et al. elucidated the role of metastasis-associated lung adenocarcinoma transcript 1 (MALAT1) in ECs. Using genetic MALAT1 knockout mice the authors showed MALAT1 is necessary for proper development of blood vessels and regulates the angiogenic features of vascular cells (317). In contrast, suppression of maternally expressed 3 (Meg3) lncRNA resulted in enhanced expression of angiogenesis-promoting genes (321) and prevented cardiac fibrosis and remodeling by reducing MMP-2 (334).

BACE1 has recently been recognized to play an important role in neurodegenerative diseases like Alzheimer's disease via regulating beta-amyloid peptide (Aβ) production (335–337). Interestingly, a recent study showed that Beta-Site Amyloid Precursor Protein Cleaving Enzyme (BACE1) and BACE1-AS expression was upregulated in left ventricle biopsies from patients with non-end stage ischemic HF (338) and in animal models of ischemic HF (318). The report demonstrated that BACE1 increased the production of beta-amyloid peptide and decreased the number of ECs and cardiomyocytes by activation of apoptosis. In a separate study using high fat diet-induced obesity in mice, Kim et al. showed that BACE1 transcriptional activity was activated through Akt-mTOR signaling in response to palmitic acid treatment resulting in increased beta amyloid peptide accumulation in neuronal cells (339). Whether similar mechanism exists in cardiomyocytes needs further investigation. Moreover, BACE1 was also found to play a role in atherosclerosis (319) and MI-induced neuro-inflammation in brain (318). Another human lncRNA, ANRIL (antisense non-coding RNA in the INK4 locus) has been associated with a locus implicated in CVD (340). ANRIL was shown to be highly upregulated in atherosclerotic plaques in patients with genetic polymorphism in chromosome 9p21 locus, which overlaps with ANRIL coding region (322–324, 340). A recent study suggests that the higher expression level of ANRIL is associated with presence of CAD in diabetic patients and could be considered as a potential peripheral biomarker (325).

According to reports, the smooth muscle and EC-enriched migration/differentiation associated long non-coding RNA (SENCR) was downregulated in VSMCs of diabetic mice and enhanced VSMC proliferation and migration through induction of FOXO1 and short transient receptor potential Channel (TRPC6), a target of SENCR (31, 341). SENCR is also recognized as a strong circulating biomarker for left ventricular dysfunction in type 2 diabetes (316). In line with this, smooth muscle-induced lncRNA enhances replication (SMILR) was also shown to be highly upregulated in unstable atherosclerotic plaques and in plasma of patients with increased plasma C-reactive protein levels (326). Further, knockdown of SMILR in primary human saphenous vein-derived endothelial cells (HSVECs) treated with IL-6/PDGF reduced proliferation (326). Another interesting lncRNA, E330013P06, is upregulated in macrophages from db/db and diet-induced insulin-resistant type 2 diabetic mice and in monocytes of diabetic patients (329). It is also increased along with inflammatory genes in mouse macrophages treated with high glucose and palmitic acid. Overexpression of E330013P06 in macrophages induces the expression of pro-inflammatory and pro-atherogenic genes, which leads to enhanced inflammatory signals and foam cell formation. Silencing E330013P06 was shown to reverse the upregulation of inflammatory genes induced by diabetes (342). Another lncRNA, MeXis, was identified to play a role in macrophage cholesterol efflux and atheroegenesis (343). A causal link between liver X receptors (LXRs), sterol-activated nuclear receptors that regulate the expression of genes involved in cholesterol homeostasis, and MeXis was established by Sallam et al. (343). The study showed that MeXis and ABCA1 expression were induced by LXR in macrophages. MeXis knockout mice displayed decreased expression of Abca1 in heart and enhanced development of atherosclerosis. Induction of MeXis expression in response to activation of LXRs augmented Abca1 expression and macrophage cholesterol efflux.

A recent clinical study identified two cardiac-specific/relevant circulating lncRNAs, namely NRON (non-coding repressor of NFAT) and MHRT (myosin heavy-chain-associated RNA transcripts), which are significantly elevated in plasma of HF patients as compared to healthy participants (263). The study suggested that circulating levels of NRON and MHRT may be new independent predictors for HF. Spearman's rank correlation analysis showed that NRON is negatively correlated with HDL and positively correlated with LDH (lactate dehydrogenase), whereas MHRT is positively correlated with AST (Aspartate aminotransferase) and LDH.

Interestingly, HOX (Homeobox) antisense intergenic RNA (HOTAIR), a reportedly cardioprotective lncRNA, was reduced in AMI patients and its concentration was inversely correlated with cTnI and miR-1 levels (327). Moreover, adenovirus-mediated overexpression of HOTAIR reduced hypoxia-induced apoptosis in cultured cardiomyocytes with suppression of miR-1 expression (327). Reports in the literature also indicate that the expression of HOTAIR is dramatically down-regulated in both hypertrophied heart tissues and cultured cardiomyocytes treated with Ang-II, which correlated with increased cell surface area and up-regulated expression of ANP and BNP (328). Overexpression of HOTAIR reduces the expression of pro-hypertrophic markers like ANP, BNP, and β-MHC in response to Ang-II stimulation (328). The same study also suggests that HOTAIR serves as a miR-19 sponge and functionally interacts with it. Overexpression of HOTAIR inhibits miR-19 with subsequent retrieval of the expression of PTEN (a direct target of miR-19), which is involved in HOTAIR-mediated inhibition of cardiac hypertrophy.

Taken together, although much more is known about miRNAs than lncRNAs, there are exceedingly more lncRNAs (~30,000) compared to miRNAs (~2,000). More targeted research to provide comprehensive understanding of lncRNAs, coupled with improved delivery methods will further advance the field of lncRNAs and offer more insight into their involvement in CVD before they can be used as therapeutic targets.

### Piwi-RNA

P-Element induced wimpy testis RNAs (PIWI-RNA, piRNA) are lncRNAs that interact with piwi protein family and act as RNA-guided gene regulatory elements (342, 344). They play an important role in epigenetic control of gene expression through DNA methylation, regulation of TEs, and maintenance of genome integrity (345–351). They are involved in the pathogenesis of various types of cancer (352–356) and are master regulators of inheritance. However, relatively much less is known about the functional role of piRNAs in the field of CVDs. piRNAs are generally known to interact with several TEs and silence them to maintain genome stability (63, 67). In this regard, piRNAs influence long interspersed nuclear elements (LINEs) in the heart and suppression of LINE-1 decreases ischemic damage through activation of the Akt/PKB signaling (357, 358). Similarly, global microarray analysis revealed differentially expressed piRNAs in cardiosphere and cardiosphere-derived cells. The study also reported that 181 piRNAs are upregulated and 129 are downregulated in cardiosphere-derived cells with respect to cardiac fibroblasts (357, 359). Interestingly all upregulated piRNAs targeted LINE elements and in particular, piRNAs DQ594975, DQ572313, DQ586118 activated pro-survival AKT signaling. The overall result indicated that piRNAs could play a functional role in cardiomyocyte proliferation and regeneration. Reports also speculate that piRNAs can regulate AKT signaling through interaction with PIWIL2 protein. One study showed that PIWIL2 protein is highly expressed in tumor cells. Experiments using constitutive expression of PIWIL2 in NIH-3T3 cells demonstrated that it inhibits apoptosis through activation of Stat3/Bcl-XL pathway (360). Since piwi-interacting RNAs predominantly regulate TEs, aberrant expression mostly results in gene mutations leading to cancer and genome instability in inherited genetic disorders. Nevertheless, research findings of piRNAs in heart disease, such as cardiac hypertrophy and other proliferation related abnormalities are emerging and can further enhance our understanding on the role of piRNAs in CVD. In a recent publication, Rajan et al. reported abundant expression of piRNAs in the cardiac system and altered expression profile during cardiac hypertrophy in both chronic swimming-induced hypertrophied rat heart and control rat heart *in vivo*, and alpha-2 macroglobulin-induced hypertrophied H9c2 cells and control H9c2 cells *in vitro* (264). The study identified 22 potential piRNAs showing differential expression during cardiac hypertrophy, which was validated by qPCR and by RNA immunoprecipitation using piwi antibodies for piR_2106027. Analyzing the expression of piR_2106027 in different patients with and without MI, Rajan et al. also revealed that piR_2106027 is significantly elevated in patients with MI. Interestingly, they also identified that the level of piR_2106027 correlates with cTnI levels as this piRNA was not elevated in cTnI-negative MI patients. Accordingly, piRNAs were suggested as vibrant diagnostic markers as well as potential therapeutic targets for CVD.

### Circular RNA (circRNA)

Relatively new players in the world of ncRNAs, much less is known about the role of circular RNA (circRNA, a closed continuous loop of ncRNA with 3′ and 5′ ends joined together) in CVD. However, circRNAs have gained much attention recently due to their sustained stability (361) and recognized as novel biomarkers (362, 363). CircRNAs were identified as competing endogenous RNAs (ceRNAs) that sponge specific miRNAs by complementary base pairing (364). A recent publication reported that mice with overexpression of ARC (apoptosis repressor with caspase recruitment domain) exhibit reduced hypertrophic response and that ARC is a direct downstream target of miR-223 (365). A heart-related circRNA (HRCR) acts as an endogenous miR-223 sponge and inhibits miR-223 activity, which resulted in the increase of ARC expression and attenuation of cardiac hypertrophy and HF.

Studies using mouse models of MI and isolated cardiomyocytes subjected to hypoxia showed that cerebellar degeneration-related protein 1 transcript (Cdr1as) is highly upregulated with MI and overexpression of miR-7 rescues Cdr1as-induced cardiac apoptosis (366). It was also observed that overexpression of Cdr1as induces the transcript levels of PARP and SP1 and that Cdr1as functions as a miR-7a sponge in promoting MI-related injury. Similarly, cZNF292 was identified to play a role in endothelial function and was up-regulated upon hypoxic treatment. Inhibition of cZNF292 reduced angiogenic sprouting of ECs and its overexpression increased endothelial proliferation (367). Several circRNAs were implicated as novel biomarkers in MI. A recent study using blood samples from 472 patients with acute MI showed MICRA as a novel biomarker for risk classification of MI patients (265). The study analyzed the correlation between the expression of MICRA to patient group to reduced EF (rEF), mid-range EF (mrEF), and preserved EF (pEF). Interestingly the data suggest that MICRA could be a significant predictor of LV dysfunction in patients after acute MI. A recent clinical study identified another circRNA, hsa_circ_0124644, which was remarkably increased in the peripheral blood of 137 patients with CAD compared to control subjects, suggesting that it may be a promising diagnostic biomarker (363). Gupta et al. identified specific circRNAs derived from Ttn (Titin), Fhod3 (Formin homology 2 domain containing 3), and Strn3 (Striatin, calmodulin-binding protein 3), which are altered in the heart upon doxorubicin treatment (368). Knockdown of Ttn-derived circRNA was shown to enhance the susceptibility to doxorubicin, elucidating the functional role of circRNAs in doxorubicin-induced cardiotoxicity.

### Therapeutic approach: current and future perspectives and controversies

Several promising therapeutic outcomes in animal models and pre-clinical trials have encouraged pharmaceutical companies to conduct ncRNA-based clinical trials in patients with CVD and other diseases. MRG-201, an anti-fibrotic synthetic miRNA mimic (promiR) of miR-29b, is designed to decrease the expression of collagen and reduce fibrotic scar in diseases like idiopathic pulmonary fibrosis and recently completed its phase I clinical trial in healthy volunteers to evaluate its tolerability (Miragen Therapeutics, Inc.). Miragen also has several miRNA-based pipeline projects including MRG-110, which targets miR-92a to enhance the revascularization process in ischemic heart disease. MGN-1374 (miR-15 family) to treat MI and MGN-9103 (miR-208) for the treatment of obesity and diabetes are also among the novel potential therapeutic candidates developed by Miragen. LNA-antimiR-34a, which targets Notch1 and Sema-4b (104, 369), is also being considered for the treatment of MI by miRNA Therapeutics, Inc. Several other miRNA-based therapies that are currently in clinical trials can be found on the National Institutes of Health (NIH) website (https://clinicaltrials.gov/). NcRNA-based therapies to treat CVD in clinical trials are very limited and are still in the developmental stage. However, RNA-based clinical trials, especially with siRNA and miRNA to treat devastating diseases like cancer (370–372) have shown promising results and are therefore encouraging to extend similar approaches to treat CVDs.

Although the path to ncRNA-based therapy looks promising, it is also fraught with limitations. The fact that a single miRNA can target several mRNAs, which is considered an advantage to exert broad effects on multiple pathological pathways can also be viewed as a major limitation that may evoke undesired responses. Moreover, a critical issue with ncRNA-based therapy is delivery of miRNA mimics/antagomiRs to the target organ while withstanding degradation in the blood stream. Researchers have tried several approaches to circumvent this problem such as adhesive hydrogel patch delivery (373), nanoparticle-mediated delivery (371, 374), exosome-mediated delivery (375, 376), and antibody-fused nanoparticles (377). Important considerations to improve miRNA based therapy include enhancement of target affinity, stability, specificity and ADME (absorption, distribution, metabolism, and excretion) (378). Several chemically modified oligonucleotides either antagonizing or mimicking miRNAs have been efficiently used in preclinical models (379–381). These synthetic oligonucleotides possess better stability, absorption in the cell and offer greater affinity toward their target (382, 383). The efficacy of these novel oligonucleotide-based drugs has accelerated clinical trials to target miRNA-based gene therapy. Several classes of oligonucleotide drugs with unique modifications in their structure have enhanced their pharmacokinetics, metabolism, stability, resistance against nuclease activity in the blood stream and allowed for better bioavailability (380, 384).

Since exonucleases cleave the phosphodiester bond, a substitution of phosphodiester group with phosphorothioate in the nucleotide backbone and replacing the ribose sugar moieties with 2′-O-methyl or other 2′s confer substantial resistance toward nuclease activity (381, 385, 386). Antisense oligonucleotides (ASO) are first generation miRNA inhibitors, with increased oligonucleotide stability but less binding affinity toward target RNA due to the presence of phosphorothioate. However, this obstacle was overcome with the use of 2′-O-methyl (2′-OMe) modification of RNA (2′-OMe-RNA), which possesses both improved binding affinity and nuclease resistance (387–389). Among the ASOs, 2′-O-methoxyethyl (2′-MOE) is the most successful and widely used oligonucleotide inhibitor, with increased nuclease resistance and substantially higher binding affinity than its predecessors (390, 391). Several other modifications, such as 2′-fluoro (2′F), which further increases the affinity of oligonucleotides also proved effective (392, 393). Locked nucleic acid (LNA) is another modified RNA oligonucleotide that has an additional bond between the 2′ oxygen and 4′ carbon that was shown to have increased binding affinity for its target (394–396). LNA-based miRNA inhibition has the advantage over other chemically modified oligonucleotides by interfering in the alternative gene splicing (397) and is also widely used in pharmaceutical industry (398). Indeed, the first miRNA-targeted drug tested for safety in phase II clinical trial of HCV infection was based on LNA chemistry (399). Several other chemically modified ASOs, such as aptamers, single-stranded oligonucleotides that bind to the protein and disrupt the protein-protein interaction (400), and LNA modified with constrained ethyl' (cEt) (401) and Ribonuclease H1-dependent LNA-Gapmers (402) have also been investigated with moderate success. All these methodologies have been used with considerable success in animal models thus far and are promising for further development. Novel delivery methods using AAV vectors guided with cardiac specific promoters such as α-MHC or troponin can also be considered when translating these findings to human subjects. Emerging revolutionary genome editing techniques like Clustered Regularly Interspaced Short Palindromic Repeats (CRISPR-CAS9) are already considered for clinical trials to correct genetic disorders like β-thalassemia (372), cystic fibrosis transmembrane conductor receptor (CFTR) mutation (373), and Duchenne muscular dystrophy (374) (DMD). CTX001 is a new initiative by European Union and United States Food and Drug Administration (FDA) for CRISPR-based treatment of β-thalassemia, which is expected to start in 2018. Exploiting CRISPR/CAS9 technology to edit miRNA and other ncRNAs is also a novel option and demonstrated to be viable for stable suppression of miRNAs (403). Nevertheless, the scientific community should approach these new treatment strategies with extreme caution to avoid off-target effects.

In spite of its therapeutic potential, miRNA-based gene therapy is also challenged with controversies. One such example is miR-21, where antagomir-mediated inhibition of miR-21 was protective against pressure overload-induced cardiac stress (91), yet genetic ablation or LNA-based inhibition of miR-21 in mice failed to protect against TAC (404). These discrepancies between studies caution us to consider the efficiency of inhibition when using different types of inhibitors, such as LNA- or cholesterol-based antagomir. In addition, the non-specificity of blocking multiple miRNAs that have similar complementary sequences in the seed region may contribute to the functional outcome. Genetic knockout and antagomir-based inhibition of miR-143/145 demonstrated different response to stress in VSMCs (405, 406). Ablation of miR-143/145 blocked the induction of VSMC proliferation and developed neointimal lesions (405, 406). In striking contrast, miR143/145 knockout mice displayed reduced neointima formation in response to carotid artery ligation (407). However, these studies investigated different targets of miR-143/145 that are involved in cytoskeletal regulation (405–407). Therefore, these controversial outcomes, either due to the redundancy of miRNA targets or the types of methods used for miRNA inhibition, warrant further in-depth studies.

## Conclusion

The robust development and the application of ncRNAs in myriad fields has opened up venues for many novel research areas such as precision and personalized medicine and may offer a unique class of biomarkers. The powerful role of ncRNAs in defining cell fate both in normal and pathological states has provided an opportunity to device new therapeutic strategies to combat CVD. Recent preclinical and clinical studies suggest that ncRNA-based gene therapy is not too far from clinical trials. However, despite the recent surge in the field of ncRNAs, our understanding about the function of these small molecules remains rather limited. Future studies are warranted to shed new light on the involvement of ncRNAs in CVD so we may be able to utilize them as biomarkers and/or target them to carefully regulate cell function for treatment of CVD and its risk factors.

## Author contributions

AS, AD, and FS drafted, edited, and approved the manuscript and figures.

### Conflict of interest statement

The authors declare that the research was conducted in the absence of any commercial or financial relationships that could be construed as a potential conflict of interest.
